# Large‐Area Blade‐Coated Deep‐Blue Polymer Light‐Emitting Diodes with a Narrowband and Uniform Emission

**DOI:** 10.1002/advs.202205411

**Published:** 2022-12-27

**Authors:** Shengjie Wang, Lili Sun, Yingying Zheng, Yahui Zhang, Ningning Yu, Jinghao Yang, Mengyuan Li, Wenyu Chen, Liangliang He, Bin Liu, Mingjian Ni, Heyuan Liu, Man Xu, Lubing Bai, Jinyi Lin, Wei Huang

**Affiliations:** ^1^ School of Flexible Electronics (Future Technologies) (SoFE) and Institute of Advanced Materials (IAM) Nanjing Tech University (NanjingTech) 30 South Puzhu Road Nanjing 211816 China; ^2^ School of Materials Science and Engineering Institute of New Energy College of Science China University of Petroleum (East China) Qingdao Shandong 266580 China; ^3^ State Key Laboratory of Organic Electronics and Information Displays & School of Chemistry and Life Sciences Nanjing University of Posts & Telecommunications 9 Wenyuan Road Nanjing 210023 China; ^4^ Frontiers Science Center for Flexible Electronics (FSCFE) Shaanxi Institute of Flexible Electronics (SIFE) & Shaanxi Institute of Biomedical Materials and Engineering (SIBME) Northwestern Polytechnical University Xi'an 710072 China

**Keywords:** blade‐coating processing, large‐area deep‐blue polymers light‐emitting diodes, polydiarylfluorenes, single‐chain emission behavior, uniform deep‐blue emission

## Abstract

Large‐area polymer light‐emitting diodes (PLEDs) manufactured by printing are required for flat‐panel lighting and displays. Nevertheless, it remains challenging to fabricate large‐area and stable deep‐blue PLEDs with narrowband emission due to the difficulties in precisely tuning film uniformity and obtaining single‐exciton emission. Herein, efficient and stable large‐area deep‐blue PLEDs with narrowband emission are prepared from encapsulated polydiarylfluorene. Encapsulated polydiarylfluorenes presented an efficient and stable deep‐blue emission (peak: 439 nm; full width at half maximum (FWHM): 39 nm) in the solid state due to their single‐chain emission behavior without inter‐backbone chain aggregation. Large‐area uniform blade‐coated films (16 cm^2^) are also fabricated with excellent smoothness and morphology. Benefitting from efficient emission and excellent printed capacity, the blade‐coated PLEDs with a device area of 9 mm^2^ realized uniform deep‐blue emission (FWHM: 38 nm; CIE: 0.153, 0.067), with a corresponding maximum external quantum efficiency and the brightness comparable to those of devices based on spin‐coated films. Finally, considering the essential role of deep‐blue LEDs, a preliminary patterned PLED array with a pixel size of 800 × 1000 µm^2^ and a monochrome display is fabricated, highlighting potential full‐color display applications.

## Graphical abstract

Large‐area blade‐coated deep‐blue polymer light‐emitting diodes (PLEDs) are fabricated based on encapsulated polydiarylfluorenes. Large‐area blade‐coated film presents a robust deep‐blue emission, associated with their intrachain singlet excitonic behavior. Large‐area deep‐blue PLEDs are obtained with a narrow‐band emission of FWHM ≈ 38 nm and a CIE of (0.153, 0.067), which are applied in the patterned pixel array and monochrome display.

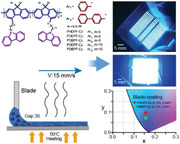

## 1 Introduction

**Figure 1:**
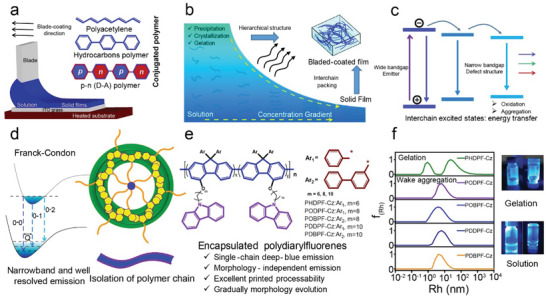
a) A schematic illustration of a printed film of the conjugated polymer via the blade‐coating. b) Fluid mechanical phenomena of conjugated polymers solution along blade‐coating directions. c) Undesirable interchain excited state formation caused by the precipitation, crystallization, and gelation in the blade‐coating. d) Self‐encapsulated strategy to suppress free rotation, vibrational splitting, and intermolecular aggregation without excimer emission in the solid state with the aim of inducing narrowband emission. e) Design and preparation of our encapsulated polydiarylfluorene toward large‐area blade‐coated PLEDs. Long lengths of side chain at 4‐position and large steric units at 9‐position can yield efficient and stable deep‐blue emission with a single‐chain emission while retaining solution processability and favorable behavior during morphology evolution. f) DLS curves of four polymers and PHDPF‐Cz in toluene soluiton (0.5 mg mL^−1^, Left), together with the photographs of their interchain behavior in solution state under UV 365 nm lamp (Right). Short length of side chain in reported PHDPF‐Cz results in a strong gelation behavior under extremely concentrated states (Right, top ones), which may cause rapid and serious precipitation and gelation in blade‐coating processing. These problems are easily resolved for the other four polymers (Right, and bottom ones). PHDPF‐Cz/toluene gel was obtained (top) but a flowable solution for other four polymers (below).

Emerging polymer light‐emitting diodes (PLEDs) have attracted considerable interest from both academics and industrial scientists owing to their high emission efficiency, potential mechanical flexibility, large‐scale solution fabrication, low cost, and facile structural modification.^[^ ^1^ ^]^ Departing from small‐area devices that have experienced great technological progress, the fabrication of large‐area PLEDs by mass‐production printing is required for low‐cost flat‐panel solid lighting and displays.^[^ ^1^, ^2^ ^]^ The manufacturing capacity for large areas by spin‐coating is low, dramatically increasing PLED costs.^[^ ^3^ ^]^ Compared to other emitters, the interpenetration and entanglement of light‐emitting conjugated polymer (LCPs) chains may enable the fabrication of large‐scale layers via printing.^[^ ^3^, ^4^ ^]^ To date, a series of mass‐production printing techniques for LCPs have been introduced for large‐scale PLED fabrication, including screen printing, slot‐die coating, inkjet printing, gravure printing, and blade‐coating, owing to their intrinsic rheological behavior.^[^ ^3^, ^4^, ^5^ ^]^ In particular, blade‐coating is an attractive technique because it does not directly contact the substrate layer, allows for high throughput and low material losses, and is a simple and convenient method to construct large‐area and uniform films.^[^ ^3^, ^6^ ^]^ In addition, blade‐coating can deposit multiple layers for optoelectronic devices due to the rapid deposition by a relatively fast‐moving blade.^[^ ^1^, ^3^ ^]^ This technique was used to fabricate all‐printed PLEDs, organic field‐effect transistors, and organic solar cells (**Figure** **1**a).^[^ ^1^, ^6^, ^7^ ^]^ Nevertheless, compared to spin‐coating, non‐uniform thickness and uncontrollable morphology along the blade‐coating direction represent major obstacles to fabricating high‐performance and stable PLEDs, although they can be partially resolved by optimizing preparation conditions.^[^ ^7b,c^ ^]^ A unidirectional nature was observed induced by the blade‐coating, which is common in meniscus‐guide coating techniques.^[^ ^4^ ^]^ This introduces some problems, including the process of thin‐film formation downstream close to the drying front that establishes a dramatic concentration gradient within the meniscus.^[^ ^4a^ ^]^ Surface tension and temperature gradients also arise along the meniscus, inducing complex flow patterns. Simultaneously, the capillary flow, Marangoni effect, and temperature gradient can cause opposite direction flows, resulting in more complex relationships.^[^ ^4b^ ^]^ The effect of their combination is difficult to predict and control, and expierments must be repeated many times to determine the best parameters for regulation. In addition, the LCP emissive layer fabricated by blade‐coating always exhibits high roughness and poor continuity due to the coffee ring effect, fast precipitation, and crystallization upon the relatively rapid solvent evaporation,^[^ ^3^, ^4^, ^8^ ^]^ which also reduces the batch ability, luminous uniformity, and color purity of large‐area PLEDs (Figure 1b). Therefore, the synergetic effects of LCP material selection and process optimization are essential. Inevitably, the hierarchical structure of blade‐coated films that governs these photophysical processes must be considered.

In large‐area PLED displays and solid light, three fundamental colors (red, green, and blue) are used to generate all other derivative colors by proportion control.^[^ ^1h^ ^]^ Specifically, the complicated morphology of the blade‐coating prepared film includes uncontrollable thickness and interchain arrangements, together with interchain *π*–*π* interactions in LCP materials,^[^ ^4b^ ^]^ generating complicated continuous processes from charge transport to the photophysics of neutral excited species.^[^ ^1^, ^9^ ^]^ Compared to the uniform small‐area films obtained via spin‐coating, the solvent evaluation during blade‐coating promotes self‐aggregation and dense packing, inducing interchain *π*–*π* electron coupling and multi‐chain excited state formation. These may result in a “guest” defect structure with a distributed narrow band‐gap, low photoluminescence (PL) efficiency, color impurity, and poor spectral stability of the deep‐blue emission (wide band‐gap; Figure 1d).^[^ ^9^, ^10^ ^]^ High‐bandgap excitons in deep‐blue LCPs are easily trapped by defect centers.^[^ ^11^ ^]^ Generally, this complex photophysical processing slightly affects the color purity, luminescence uniformity, and performance of large‐scale red and green PLEDs because of their intrinsic narrow bandgap, but these are extremely undesirable for the fabrication of large‐area deep‐blue PLEDs (Figure 1c).^[^ ^12^ ^]^ As expected, a large number of efficient and stable red, green, or sky‐blue LEDs, including organic small molecule light‐emitting diodes, quantum LEDs, and PLEDs,^[^ ^1^, ^3^, ^5^, ^8^, ^13^ ^]^ have been successfully fabricated via blade‐coating. However, stable and efficient deep‐blue LEDs (peak <440 nm) with narrowband emission have been rarely reported over the last decade.^[^ ^3^, ^13^ ^]^ Therefore, it is a serious challenge to not only obtain uniform and large‐area blade‐coated film but also completely suppress the effect of the morphology on the excitonic behavior of LCPs for deep‐blue PLEDs, which are the two key parameters to obtain high‐quality emission efficiency and uniformity. Therefore, a series of encapsulated polydiarylfluorenes (PODPF‐Cz, POBPF‐Cz, PDDPF‐Cz, and PDBPF‐Cz) were prepared herein to obtain stable and efficient single‐chain emission behavior from blade‐coated films towards the developed of large‐area deep‐blue PLEDs (Figure 1e). In our previous work,^[^ ^4a^ ^]^ we creatively proposed a facile and universal strategy to suppress the infamous Coffer‐ring Effect in droplet evaporation by introducing planar carbazole (Cz), which is an active supramolecular synthon to obtain dynamical cross‐linked frameworks, to the end‐capping group of the 4‐position pendant side chain (Figure 1d). The intense and weak *π*–*π* stacking interactions among the Cz units of encapsulated polydiarylfluorenes side chains, lead to the incensement of its solution viscosity, and hence prevent the outward flowing of the individual polymer chains and packed aggregates to the solution droplet and layer edge. In contrast to the constant viscosity of conventional LCPs solution, our encapsulated polymers exhibited distinct rheological properties and uniformly deposited on the substrate. Therefore, uniform deep‐blue emission was observed for the large‐area blade‐coated films with single‐chain emission behavior. After taking the uniform film as an emissive layer, the PLEDs exhibited deep‐blue emission with an FWHM of 38 nm and CIE of (0.153, 0.067).^[^ ^12^ ^]^ At an emission area of 4 mm^2^, the external quantum efficiency (EQE) reached 1.54%. Uniform deep‐blue emission was also demonstrated with an ultra large area of 900 mm^2^. Finally, the preliminary patterned PLED pixels and monochrome display were fabricated based on the newly prepared large‐area blade‐coated PLEDs.

## 2 Results and Discussion

### 2.1 Synthesis and Structural Characterization of Encapsulated Polydiarylfluorenes

Generally, the solution processability of LCP‐printed films is closely related to their physicochemical properties and processing parameters. Solvent selection is a key parameter that can optimize the ink viscosity and control the interchain reorganization and arrangement during film formation.^[^ ^4^, ^14^ ^]^ However, their relatively poor solubility in non‐halogenated solvents results in unfavorable aggregation, time‐dependent viscosity changes, potential rapid gelation, precipitation, and crystallization upon solvent evaporation.^[^ ^4b^ ^]^ Therefore, optimizing LCP structures to simultaneously obtain robust deep‐blue emission and retain solution processability is a delicate process but a prerequisite for fabricating high‐performance and stable deep‐blue PLEDs via mass‐production printing. Previously, self‐encapsulated polyfluorene (PHDPF‐Cz) was designed and prepared for high‐performance deep‐blue PLEDs.^[^ ^2^, ^4^ ^]^ However, it also exhibits rapid gelation in nonpolaron toluene solutions, resulting in a rough film morphology and preventing uniform emission and stable color purity.^[^ ^2^, ^8^ ^]^ To improve the processability of the printed solution, four novel encapsulated polydiarylfluorenes with long side chains were synthesized herein to manufacture stable and narrow‐band blade‐coated deep‐blue PLEDs. First, an octyl or decane side chain with a carbazole at the 4‐position was introduced for entangled attraction, and bulky diphenyl or biphenyl at the 9‐position for steric hindrance.^[^ ^15^ ^]^ The molecules were named PODPF‐Cz, POBPF‐Cz, PDDPF‐Cz, and PDBPF‐Cz (Figure S1, Supporting Information), and the original materials PHDPF‐Cz and PODPF without carbazole functional groups were used as controls. Four new polymers were prepared by traditional Yamamoto coupling polymerization and the chemical structures of the monomers and polymers were confirmed by NMR spectral analyses (Figures S2–S5, Supporting Information). In addition, the number‐average molecular weight (*M_n_*) and polydispersity index (PDI) were determined by gel permeation chromatography (GPC), yielding values of ≈2.3 × 10^4^, 2.2 × 10^4^, 2.5 × 10^4^, 2.3 × 10^4^, and 2.5, 3.1, 2.1, 2.0 for PODPF‐Cz, POBPF‐Cz, PDDPF‐Cz, and PDBPF‐Cz, respectively. A high decomposition temperature (*T*_d_) was observed for all polymers of >400 °C. Elongating the alkyl chain decreased the glass transition temperature and the free volume increased with increasing side chain group volume and increasing steric resistance at the 9‐position. The test data (Figure S6, Supporting Information) of *T*_g PODPF‐Cz_, *T*_g PDDPF‐Cz_, *T*_g POBPF‐Cz,_ and *T*_g PDBPF‐Cz_ showed that a low *T*g indicates better chain flexibility. Compared to the other four polymers, PHDPF‐Cz exhibited relatively strong interchain gelation. DLS was used herein to explore the details of the translational motion.^[^ ^16^ ^]^ Compared to the strong interchain gelation of PHDPF‐Cz in toluene solution, the lower intensity correlation function was observed and transferred to a relatively short time for PODPF‐Cz, POBPF‐Cz, PDDPF‐Cz, and PDBPF‐Cz (Figures 1f and S7, Supporting Information). Their significantly different relaxation times and scattered clusters in the corresponding graph showed that the PHDPF‐Cz chains presented the strongest physically cross‐linked aggregation in toluene. The slightly lower degree of aggregation and shorter relaxation time corresponded to good solution solubility of the material. More homogeneous solutions are conducive for the preparation of large‐area devices via printed processing.

### 2.2 Efficient and Stable Deep‐Blue Emission of Encapsulated Polydiarylfluorene in Solid States

**Figure 2:**
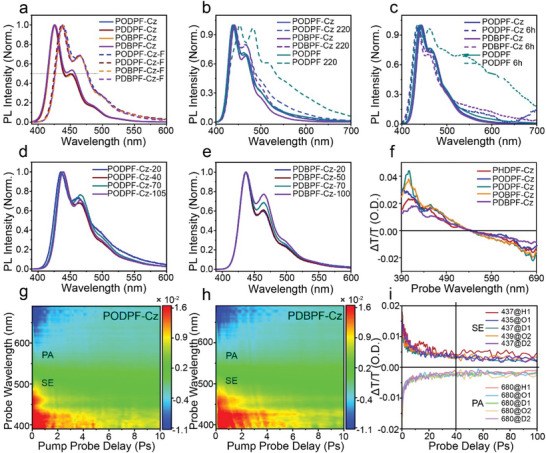
Optical properties of the four encapsulated polydiarylfluorenes in various states. a) PL spectra of PODPF‐Cz, PDDPF‐Cz, POBPF‐Cz, and PDBPF‐Cz in solutions and solid films. b) PL spectra of corresponding annealed film of PODPF‐Cz, PDBPF‐Cz, and PODPF obtained under thermal treatment at 220 °C. c) PL spectra of PODPF‐Cz, PDBPF‐Cz, and PODPF aged films. Aged films were prepared by keeping them under a UV (365 nm) lamp in an ambient atmosphere several times. d,e) PL spectra of PODPF‐Cz and PDBPF‐Cz pristine films with varying thicknesses coated from toluene solution. f) Corresponding Δ*T*/*T* spectra and i) Δ*T*/*T* kinetics of PHDPF‐CZ, PODPF‐Cz, PDDPF‐Cz, POBPF‐Cz, and PDBPF‐Cz films. g,h) TA contour plots of PODPF‐Cz and PDBPF‐Cz.

In the self‐encapsulated polymer, the pendant *π*–*π* stacking interaction of Cz units has been reported previously,^[^ ^1^, ^2^ ^]^ and the newly prepared polymers basically retained the performance advantages imparted by the original functional group. To explore their photophysical properties, optical analysis was performed to examine the excitonic behavior in various states. For example, in PODPF‐Cz, the PL spectrum of the PODPF‐Cz‐coated film consists of three well‐resolved emission peaks at 437, 464, and 496 nm attributed to the intrachain 0–0, 0–1, and 0–2 vibronic transitions of polyfluorene, respectively. A similar PL spectral profile with a slight redshift of ≈11 nm compared to that of the diluted solution was observed for the PODPF‐Cz film (**Figure** **2**a), confirming the single‐chain emission behavior in the solid state.^[^ ^9^, ^17^ ^]^ This slight red shift was attributed to the enhanced molecular conjugation due to the action of the solvent and the inevitable quenching aggregation in the film state. Similar emission peaks were observed for the other three polymers (Figures 2a and S8, Supporting Information). In addition, the solid film showed ultrastable deep‐blue emission behavior because of its excellent chemical stability and extremely weak interchain *π*–*π* electron coupling. When stored at 220 °C for 10 min, green band emission was clearly observed for the PODPF films and all four polymer films showed a similar PL spectral profile without a green band and presented robust deep‐blue emission (Figures 2b and S9, Supporting Information). In addition, the pristine coated films were aged in air for 3 and 6 h under UV irradiation. As expected, a significant change occurred in the PODPF film, with the appearance of additional green emissions. After 6 h, two significant bulges appeared at 483 and 517 nm (Figures 2c and S10, Supporting Information). Notably, all films prepared from the encapsulated polydiarylfluorenes showed excellent emission stability. Under the same aging conditions, no obvious defects in green emissions were observed. PODPF‐Cz, POBPF‐Cz, PDDPF‐Cz, and PDBPF‐Cz exhibited similar performance as PHDPF‐Cz, and better than that of PODPF. All aged films showed a slight blue shift, possibly due to environmental conditions.

**Figure 3:**
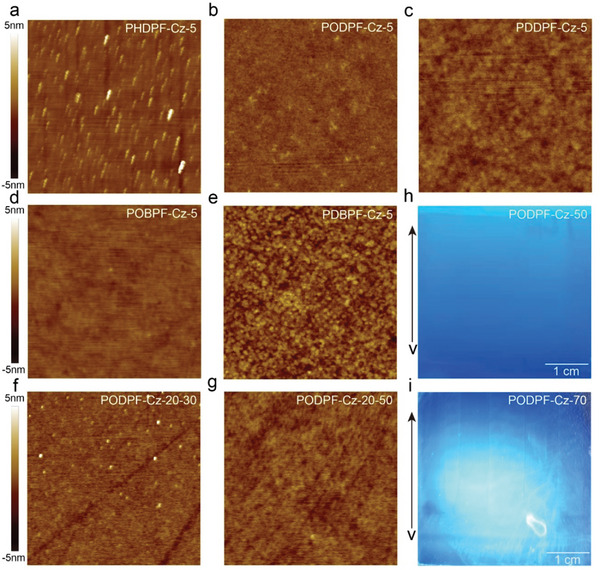
Surface morphology of the large‐area blade‐coated films of four encapsulated polydiarylfluorenes. a‐e) AFM images of different blade‐coated films: PHDPF‐Cz, PODPF‐Cz, PDDPF‐Cz, POBPF‐Cz, and PDBPF‐Cz. The scale bar is ≈5 × 5 µm. f,g) AFM images of PODPF‐Cz blade‐coated films at different temperatures of 30 °C and 50 °C of 20 × 20 µm. Photographs of blade‐coating PODPF‐Cz films in different temperatures of 50 °C h) and 70 °C i) of 4 × 4 cm^2^. (All films were prepared under the optimal process conditions, only temperature is a variable.)

As discussed above, large‐area emissive layers fabricated via printing are uniform and stable in practical applications, which require the materials to have slight morphology (such as film thickness) dependence in the luminescence process and obtain a more uniform surface morphology. To explore the influence of aggregation on optical properties in printed processing (Figure 1b), a gradient concentration solution was used to prepare films with different morphologies (thicknesses). The PL spectral shapes of the PODPF‐Cz and PDBPF‐Cz films changed slightly with increasing thickness from 20 to 100 nm (Figures 2d–e and S11–S12, Supporting Information). Even with the increased concentration, the aggregation degree showed an increasing trend with the 0–1 peak remaining lower than the 0–0 peak, which ensured the color purity of the deep‐blue emission for the printed films. Because of the inherent material properties, the change in film thickness is bound to affect the self‐absorption effect, but this problem can be overcome by choosing the appropriate concentration. This thickness‐insensitive emission property, which applies to industrial printed manufacturers, can be used to enhance the batch reproducibility of large‐area PLEDs. Meanwhile, the smooth and uniform morphology of the film surface was clearly observed using AFM (**Figure** **3**a–e). These results indicate that the newly prepared materials with carbazole showed remarkable advantages over those without carbazole in terms of the spectral stability of the resultant films. Photostability can be significantly improved by self‐encapsulation if good spreading and uniformity is simultaneously achieved, allowing for the preparation of large‐area and stable PLEDs.

Subsequently, ultrafast transient absorption spectroscopy was used to characterize the exciton dynamics of the novel encapsulated polydiarylfluorene in its film state. The spectral characteristics of stimulated emission (SE) and photoinduced absorption (PA) of the coated solid films were measured (Figures 2g–h and S15, Supporting Information). All materials exhibited strong SE bands, indicating their emissive capabilities and high exciton utilization. In addition, the differential transmission spectra were measured and analyzed (Figure 2f). Although the side chain structure lengths of the materials changed, the junction of the PA and SE signals remained static, suggesting only a slight effect. The stimulated emission (SE, Δ*T*/*T* > 0) and excited state absorption (PA, Δ*T*/*T* < 0) of several different materials were also compared. Compared to the previously reported conjugated PHDPF‐Cz at the same delay time and wavelength, the SE signal value of PHDPF‐Cz was stronger. This indicates that the signal of the novel material decayed faster and remained stable after 40 ps (Figure 2i). Concurrently, the PA signal caused by the polaron in the material decayed to a constant value very quickly, reducing exciton dissociation and yielding high fluorescence quantum efficiency. Therefore, the four encapsulated polydiarylfluorenes presented robust deep‐blue emission. The PL quantum yields (Φ) of PODPF‐Cz, POBPF‐Cz, PDDPF‐Cz, and PDBPF‐Cz films were ≈40.0%, 48.3%, 46.0%, and 60.3%, respectively (Figure S14, Supporting Information). Moreover, the lifetimes of the materials fell within a reasonable range (Figure S13, Supporting Information). Therefore, the novel encapsulated polydiarylfluorene presented intrachain singlet exciton behavior without obvious interchain aggregation in the solid state, which is essential for obtaining efficient deep‐blue emission with excellent color purity.

### 2.3 Morphology of the Encapsulated Polydiarylfluorene Blade‐Coated Films

The morphology of printed solid films is closely associated with the optical properties of the LCPs and processing parameters. Through the above analysis, encapsulated polydiarylfluorenes with a Cz unit presented robust and thickness‐insensitive deep‐blue emission. During blade‐coating, a meniscus forms between the solution and substrate, and the LCP chain aligns along the coating direction. A variety of complex interactions ultimately determine the film morphology, which must be explored at a relative equilibrium point. As expected, the coated films exhibited flat surfaces, good film‐forming properties, and uniform film thickness, and resisted crystallization and aggregation. Calibration of the process parameters was used to achieve these purposes. The encapsulated polydiarylfluorenes were proven suitable for large‐area printed processing. From the AFM surface topography, it is clear that no obvious nano‐aggregates formed in the blade‐coated films of the four novel encapsulated polydiarylfluorenes, achieving the desired effect (Figure 3a–e). For the preparation of printing inks, solvent selection is very important for controlling their rheological and interchain self‐organization behaviors. Halogenated reagents usually exhibit good solubility, but their high toxicity causes serious harm to the environment, whereas toluene exhibits relatively low toxicity and can dissolve novel polymers. Subsequently, we explored the film thickness and morphology at different coating speeds, concentration and substrate temperature (Tables S1–S4, Supporting Information). To ensure acceptable equipment performance, a 10 mg mL^−1^ toluene solution was selected before debugging the process parameters. In addition, the blade‐coating temperature was varied at 30, 50, and 70 °C (Table S1, Supporting Information). For AFM characterization, the test area was 20 µm × 20 µm to explore a wider range. At the relatively low temperature of 30 °C, the uneven film surface gathered and bulged, while small particles appeared (Figure 3f,g). At 70 °C, the higher temperature caused rapid solvent volatilization. Although no small nanoparticles were visible on the film surface during AFM characterization, the morphology was unsatisfactory at the macroscopic level. Under UV irradiation, striped lines formed along the scraping direction, indicating film thickness heterogeneity at the macro level (Figure 3h,i). The moderate temperature of 50 °C yielded better results. Next, the blade‐coating speed, gap, and amount of solution were explored to further optimize production conditions (Table S2–S4, Supporting Information). As we expected, with increasing the area of blade‐coated film, it also needs to create more volume of precursor solution on the substrates (Table S2, Supporting Information). As displayed in Table S3, Supporting Information, the film thicknesses are increasing with reducing the coated speed. Then, continuous and uniform large‐area films are achieved with a thickness of ≈40 nm under 15 mm s^−1^, which is suitable for fabricating blade‐coated PLEDs (Table S3, Supporting Information). Besides, film thickness is also reduced by decreasing the concentration of precursor ink (Table S4, Supporting Information). A significant advantage of blade‐coating over spin‐coating is the low material loss rate. When preparing films on ITO glass substrates with an area of 4 × 4 cm^2^, spin‐coating consumed at least 400–500 µL. Through extensive exploration, 80 µL was determined to be best for achieving the required film thickness. By combining these four optimized process parameters, the film thickness could be successfully controlled within the ideal range of 40–45 nm. Meanwhile, as displayed in Figure S16, Supporting Information, all large‐area blade‐coated films of four polymers had a smooth surface with an excellent and uniform deep‐blue emission (Figure S16, Supporting Information). Finally, a large‐area uniform blade‐coated film with the thickness of ≈40 nm (4 × 4 cm^2^) is obtained for further exploring their potential application for PLEDs.

### 2.4 Demonstration of Large‐Area and Patterned Deep‐Blue PLEDs

**Figure 4:**
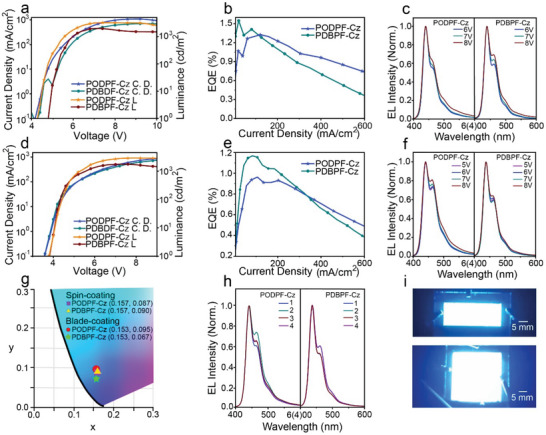
Performance of PLEDs based on encapsulated polydiarylfluorenes. a) Current density–luminance–voltage characteristics and b) External quantum efficiencies (EQE) of the PLEDs based on the PODPF‐Cz and PDBPF‐Cz spin‐coated films, together with the c) EL spectra with increasing appllied voltage. d) Current density–luminance–voltage characteristics and e) EQE curve of the PODPF‐Cz and PDBPF‐Cz blade‐coated PLEDs. f) EL spectra of the PODPF‐Cz and PDBPF‐Cz blade‐coated devices with different voltage. g) CIE of EL spectra of the PODPF‐Cz and PDBPF‐Cz spin‐coated and blade‐coated PLEDs. h) EL spectra of the blade‐coated PLEDs recorded from different positions of the same film under the same voltage. i) Photo images of large‐area blade‐coated PLEDs with different luminous areas of 1.2 × 3 cm^2^ and 3 × 3 cm^2^.

As mentioned above, the four encapsulated polymers showed thickness‐independent, efficient, and stable deep blue emission, making them suitable candidates for large‐area blade‐coated PLEDs. First, preliminary PLEDs with an ITO/PEDOT: PSS (30 nm)/emissive layer (50 nm)/TPBi (25 nm)/LiF (1 nm)/Al (100 nm) were fabricated based on their spin‐coated films (**Figure** **4**, S17–S22, Supporting Information, and **Table** **1**). As expected, the EL spectra of the four polymers exhibited two feature emission peaks at 439 (0–0) and 460 (0–1) nm, a profile similar to that of PL spectra, confirming the single‐chain emission behavior without exciton‐exciton annihilation (Figure 4c). Although there was a weak shoulder 0–1 emission at 460 nm (intensity ratio of 0–1/0–0 was ≈0.6), the device based on the PODPF‐Cz film showed a full width at half maximum (FWHM) of ≈39 nm, suggesting a narrow‐band deep‐blue emission. The FWHM of the maximum emission peak at the 0–0 band is low at ≈30 nm. The twisted main chain backbone structures of these encapsulated polydiarylfluorenes are useful for suppressing vibrational splitting, intermolecular aggregation, and free rotation in the solid state. More importantly, with increasing applied voltage (current density) from 6 to 8 V, no obvious long‐wavelength emission peaks were observed in the EL spectra of any of the devices based on the four newly developed polymers. The corresponding CIE values were (0.157, 0.087) and (0.157, 0.090) for PODPF‐Cz and PDBPF‐Cz, respectively. In addition, the PLEDs based on PODPF‐Cz and PDBPF‐Cz spin‐coated films showed luminance values of 100 cd m^−2^ at 5.7 and 5.9 V, with a maximum brightness of 4100 cd m^−2^ at 7.8 V and 1988 cd m^−2^ at 7.2 V, with corresponding turn‐on voltages (*V*_on_) were estimated at ≈4.4 and 4.6 V, respectively. The maximum EQE values of PODPF‐Cz and PDBPF‐Cz were determined to be 1.32% and 1.54%, respectively (Figures 4a,b,g, S17–S18, Supporting Information, Table 1). Therefore, stable and efficient deep‐blue emission from PLEDs without encapsulation under a high current density effectively confirmed their robust intrachain singlet excitonic behavior.

**Table 1:** Device performance of spin‐coated and blade‐coated PLEDs based on four polymers

Materials	*λ*_max_[nm]	*V*_on_[V]	*L*_max_[cd m^−2^]	EQE_max_(%)	CIE	FWHM[nm]
PODPF‐Cz (Spin‐coated)	(439, 460)	4.4	4100	1.32	(0.157, 0.087)	39
POBPF‐Cz (Spin‐coated)	(439, 462)	4.5	3146	1.32	(0.157, 0.088)	39
PDDPF‐Cz (Spin‐coated)	(438, 459)	4.5	3746	1.49	(0.153, 0.071)	38
PDBPF‐Cz (Spin‐coated)	(438, 461)	4.6	1988	1.54	(0.157, 0.090)	42
PODPF‐Cz (Blade‐coated)	(438, 462)	3.8	2904	0.96	(0.153, 0.095)	43
POBPF‐Cz (Blade ‐coated)	(439, 463)	3.8	3084	1.28	(0.156, 0.080)	46
PDDPF‐Cz (Blade‐coated)	(437, 461)	3.5	1796	0.85	(0.158, 0.085)	43
PDBPF‐Cz (Blade‐coated)	(438, 462)	3.8	1760	1.16	(0.153, 0.067)	38

PODPF‐Cz and PDBPF‐Cz were also selected as models to explore their potential application in large‐area printed deep‐blue PLEDs. Similar device configurations were applied for blade‐coated PLEDs. The device configuration of blade‐coated PLEDs is ITO/PEDOT: PSS (40 nm)/emissive layer (50 nm)/TPBi (25 nm)/LiF (1 nm)/Al (100 nm). First, large‐area uniform blade‐coated films were fabricated with an area of 3 × 3 cm. To investigate device performance, the area of the effective emission PLED cells was ≈9 mm^2^. Consistent with the above analysis, large‐area blade‐coated PLEDs exhibited efficient and uniform deep‐blue emission. Similar to the PLEDs based on the spin‐coated films, two emission peaks at 438 and 462 nm were observed for the PODPF‐Cz and PDBPF‐Cz blade‐coated PLEDs, respectively, indicating a lack of interchain crystallization and rapid random precipitation during blade coating. The narrow FWHM in the emission spectra of PLEDs based on PDBPF‐Cz films was ≈38 nm, but the weaker 0–1 band emission resulted in a better deep‐blue color purity (CIE: 0.153, 0.067) than those of devices based on the spin‐coated films (Figure 4g, Table 1). Importantly, the blade‐coated PLEDs exhibited excellent spectral stability with increasing current density. When a voltage was applied to the blade coating device, no unexpected green‐band emission was observed and the uniform luminescence indicated that our calibration and selection of film thickness were relatively accurate. To confirm the uniform deep‐blue emission, the luminescence at different positions of the large‐area PLEDs was examined and similar EL spectra with the same CIE were obtained (Figures 4h and S21–S22, Supporting Information). Although the variable film thickness and morphology of the emissive layers in different areas could not be completely avoided for large‐area printed PLEDs, uniform deep‐blue emission was easily obtained with the novel polymers due to their morphology‐insensitive deep‐blue emission. Meanwhile, large‐area blade‐coated PLEDs exhibited a performance comparable to that of devices based on spin‐coated films. The blade‐coated PLEDs with PODPF‐Cz and PDBPF‐Cz showed luminance of >100 cd m^−2^ at 5.2 and 5.2 V, with maximum brightness values of 2904 and 1760 cd m^−2^, respectively. However, both devices exhibited relatively low *V*_on_ values of 3.8 V, respectively. The corresponding maximum EQE of PODPF‐Cz and PDBPF‐Cz were estimated as 0.96% and 1.16%, respectively (Figures 4d,f, S19–S20, Supporting Information). As shown in Figure S23, Supporting Information, half‐life time of large‐area blade‐coated PLEDs based on PODPF‐Cz, PDDPF‐Cz, and POBPF‐Cz, PDBPF‐Cz are calculated ≈32 min, 44 min and 66 min, min, under the current density of ≈15 mA cm^−2^ and brightness density of 100 cd cm^−2^, 80 respectively, which is much more longer than those of PODPF ones (32 min, Figure S24, Supporting Information). Meanwhile, our novel encapsulated polymers had an excellent deep‐blue emission with a relatively stable CIE under prolonged operation time (Figures S23 and S24, Supporting Information). Therefore, large‐area blade‐coated PLEDs based on our novel polymer present a long half‐life time with an excellent spectral stability (color purity). Finally, large‐area blade‐coated films with an overall area of 4 × 4 cm^2^ were fabricated with emitting areas of 1.2 × 3 and 3 × 3 cm^2^ successfully lit, presenting a uniform and efficient deep‐blue emission (Figures 4i and S25, Supporting Information). Blade coating technology is an important process that can ensure reasonable device performance and additional possibilities.

**Figure 5:**
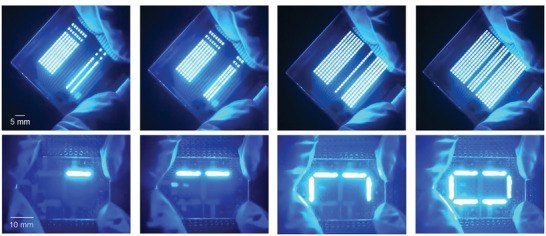
Photographic images of patterned deep‐blue PLEDs with 800 µm × 1000 µm pixels (Top). Photographs of a demo of the monochrome display based on large‐area PLEDs (Bottom).

Generally, uniform deep‐blue emission from large‐area PLEDs is required for fabricating display panels and solid films. Encouraged by the above findings, patterned PLEDs that resemble LED displays could be realized. First, large‐area PLEDs with uniform deep‐blue emissions were fabricated via blade coating (Figure S25, Supporting Information). **Figure** **5** shows a photograph of the prepared PLEDs patterns with a pixel area of 32 × 32 mm^2^ and pixel diameter of 800 µm × 1000 µm, with a bright and uniform deep‐blue emission. More importantly, each pixel exhibited uniform emission without visible defects. Meanwhile, to control the electrical current via microcontrollers, the emission intensity of all pixels was precisely tuned (Figure 5). The pixels in the two directions showed uniform and efficient deep‐blue emission with a clear and sharp edge area. Furthermore, monochrome displays based on large‐area PLEDs were fabricated with areas of ≈24 mm^2^. Similar to the results presented above, all pixels presented a uniform deep‐blue emission. All experimental results effectively confirmed the efficiency and uniformity of the large‐area blade‐coated deep‐blue PLEDs. Considering the wide potential applications of deep‐blue LEDs, it is believed that this study will promote the application of flat‐panel lighting and full‐color display PLEDs.

## 3 Conclusion

In summary, a series of stable encapsulated polydiarylfluorenes was obtained with morphology‐insensitive emission behavior for large‐area blade‐coated deep‐blue PLEDs. Compared to the reference PHDPF‐Cz, the four novels encapsulated polydiarylfluorenes presented an efficient and stable deep‐blue emission in solid films and exhibited excellent film printing capacity for large‐area uniform films via blade coating. All polymer blade‐coated films exhibited high PLQY values of ≈60% and ultra‐stable deep‐blue emission, even after aging in air under a UV lamp, confirming their robust deep‐blue emission without obvious polar formation. Large‐area and uniform blade‐coated films with excellent surface morphology and efficient deep‐blue emission were manufactured. Accordingly, PLEDs based on blade‐coated films prepared on a large area of 3 × 3 mm^2^, achieved high external quantum efficiencies of 1.16% over emitting areas of 0.09 cm^2^. Large PLEDs with an emitting area of 3 × 3 cm^2^ were also fabricated and exhibited bright and uniform emission characteristics. Finally, uniform and large‐area patterned PLED arrays with 800 µm × 1000 µm pixels and monochrome displays further confirmed the great potential of the newly developed blade‐coated PLED for display and lighting technologies. The results presented herein suggest that the encapsulated LCPs are promising candidates that can be coupled with printing (blade‐coating) methods for the production of large‐area PLEDs for lighting and flat‐panel applications due to their efficient emission and excellent processing properties.

## Conflict of Interest

The authors declare no conflict of interest.

## Supporting information

Supporting Information

## Data Availability

The data that support the findings of this study are available in the supplementary material of this article.
