# Supplementary material for: Large‐Area Blade‐Coated Deep‐Blue Polymer Light‐Emitting Diodes with a Narrowband and Uniform Emission
Source: Adv Sci (Weinh). 2022 Dec 27;10(6):2205411. doi: 10.1002/advs.202205411 (PMC9951302; doi:10.1002/advs.202205411)
Supplement: Supplementary file 1 — Supporting Information [file ADVS-10-2205411-s001.pdf]

Supporting Information

Large-area Blade-Coated Deep-Blue Polymer Light-emitting Diodes with a Narrowband and Uniform Emission

Shengjie Wang, Lili Sun, Yingying Zheng, Yahui Zhang, Ningning Yu, Jianghao Yang, Mengyuan Li, Wenyu Chen, Liangliang He, Bin Liu, Mingjian Ni, Heyuan Liu, Man Xu, Lubing Bai, Jinyi Lin* and Wei Huang**

((Optional Dedication))

Miss Shengjie Wang, Miss Lili Sun, Miss Yingying Zheng, Mr Yahui Zhang, Mr Jinghao Yang, Miss Ningning Yu, Miss Mengyuan Li, Miss Wenyu Chen, Mr Liangliang He, Miss Bin Liu, Mr Mingjian Ni, Dr Lubing Bai, Prof. Jinyi Lin and Prof. Wei Huang
School of Flexible Electronics (Future Technologies) (SoFE) and Institute of Advanced Materials (IAM)
Nanjing Tech University (NanjingTech), 30 South Puzhu Road, Nanjing 211816, China.
E-mail: iamjylin@njtech.edu.cn, wei-huang@njtech.edu.cn
Dr Man Xu, Prof. Wei Huang
State Key Laboratory of Organic Electronics and Information Displays & Institute of Advanced Materials (IAM), Nanjing University of Posts & Telecommunications, 9 Wenyuan Road, Nanjing 210023, China.
E-mail: 2399471083@qq.com
Prof. Wei Huang
Frontiers Science Center for Flexible Electronics (FSCFE), Shaanxi Institute of Flexible Electronics (SIFE) & Shaanxi Institute of Biomedical Materials and Engineering (SIBME), Northwestern Polytechnical University, Xi'an 710072, China.
Prof. Heyuan Liu
School of Materials Science and Engineering, Institute of New Energy, College of Science, China University of Petroleum (East China), Qingdao, Shandong, 266580, China.
Keywords: Large-area Deep-Blue Polymers Light-emitting Diodes, Blade-Coating Processing, Polydiarylfluorenes, Single-chain Emission Behavior, Uniform Deep-Blue Emission

EXPERIMENTAL SECTION

Chemicals

All reagents from commercial sources were used without further purification, unless otherwise noted. Anhydrous THF was pre-dried over molecular sieves. All dry reactions were performed with glassware that was flamed under a high vacuum and backfilled with N_2. Column chromatography was performed on silica gel 60 (0.040-0.063 mm). PODPF-Cz, PDDPF-Cz, POBPF-Cz, PDBPF-Cz and their monomers were synthesized according to our previous work.

General Measurement and Characterization

^{1}H and ^{13}C NMR spectra were recorded on a Bruker Ultra Shield plus 400 MHz spectrometer. The molecular weights were estimated by gel permeation chromatography (GPC) analysis using DMF as the eluent and linear polystyrene as the standard. The UV-visible absorption spectra were taken with a Shimadzu UV-1750 spectrometer at room temperature, and photoluminescence spectra were measured using the Hitachi F-4600. Thermogravimetric analysis (TGA) was acquired by TGA2 (Mettler Instruments). Differential scanning calorimetry (DSC) data was measured by DSC214 Polyma (NETZSCH Instruments) with the measured temperature from 30 to 320°C at a rate of 10 °C/min. The film morphologies of polymer films were measured with AFM in tapping mode (Bruker's Dimension Icon). The dynamic light scattering (DLS) measurements were carried out using an ALV/CGS-3. The film thickness was measured with the KlA-Tencor (P-7). Fluorescence lifetime and Photoluminescence Quantum Yield (PLQY) were measured with Time-Correlated Single Photon Counting (FLS 980).Transient absorption (TA) analysis of polymer films was measured with Nanosecond Transient Absorption Spectroscopy equipment (NANO-TA100). All the thick film were spin-coated with a speed of 1500 r/min (60 s).

Fabrication and Characterization of PLEDs

The PLED devices were prepared and characterized following the process as follows. The ITO substrates were cleaned in an ultrasonic bath with detergent, deionized water, alcohol and acetone, dried in an oven at 120°C for 2 hours, blown surface by N_2 and treated with ultraviolet ozone for 15 min before spincoating. Firstly, a 40 nm thick PEDOT: PSS was spin-coated with a speed of 1500 r/min (30 s) and then annealed at 120°C for 20 minutes. For the blade-coated device, a 150 nm thick PEDOT: PSS was blade-coated with a speed of 5 mm/s, gap was 50 μm and heated with 80°C, and then annealed at 120°C for 30 minutes. Then, the

emitting layer was spin-coated from toluene solution (10 mg/mL) with a speed of 1500 r/min (60 s) and annealed at 100°C for 15 minutes in nitrogen-filled glovebox. And the thickness of the polymer spin-coated film was estimated at about 40 nm. The emitting layer was blade-coated from toluene solution (10 mg/mL) with a speed of 15 mm/s, gap was 200 μm and heated with 50°C, and annealed at 100°C for 15 minutes in nitrogen-filled glovebox. And the thickness of polymer blade-coated film is estimated at about 40 nm too. Finally, the residue layers, such as 25 nm TPBi, 1 nm LiF, and 100 nm Al, were deposited by thermal evaporating at a pressure below 1×10^{-5} mbar. The J-L-V curves were recorded using a combination of a Keithley source meter (model 2602) and a luminance meter. The EL spectra of the devices were measured using a PR-655 spectrophotometer. All the measurements were taken in ambient conditions at room temperature.

The configuration of spin-coated PLEDs was ITO/PEDOT: PSS (30 nm)/emissive layer (50 nm)/TPBi (25 nm)/LiF (1 nm)/Al (100 nm). And the configuration of blade-coated PLEDs was ITO/PEDOT: PSS (40 nm)/emissive layer (50 nm)/TPBi (25 nm)/LiF (1 nm)/Al (100 nm). ITO is used as anode, PEDOT: PSS is used as hole-transporting layer, TPBi acts as hole-injection layer and can also play the role of partial hole-blocking layer, LiF reduces the work function of cathode playing the role in modifying Al, Al is used as cathode.

The synthesis procedures for target product

Poly[4-(6-(9H-carbazol-9-yl)octyloxy)-9,9-diphenylfluorene]-co-[5-(6-(9H-carbazol-9-yl) octyloxy)-9,9-diphenylfluorene] (PODPF-Cz) was synthesized according to our previous report about PHDPF-Cz.

First, fully dried a Shrek flask (25 mL) cleaned in advance, then degassed with N_2 five times, when it had cooled to room temperature, wrapped it with tin foil and kept it away from light for standby. Then put bipyridine (0.5 g) into a Shrek flask quickly, add $Ni(COD)_2$ (0.5 g) in dark, degassed with N_2 for five times again. Next, add COD (0.3 mL) slowly, followed by adding the dry deaeration DMF (5 mL) into the reaction tube and activate it under the condition of 75°C away from light for 30 min.

Under N_2 protection, the prepared PODPF-Cz (0.5 g) sample was dissolved in dry deaeration toluene (10 mL). Inject the sample solution into the Shrek flask and raise the temperature to 85°C under N_2 atmosphere equipped with a magnetic stir bar at dark for 3 days. At the end of the polymerization, bromobenzene (0.3 mL) was added to the flask and refluxed for 1 day. After the reaction, the solution was cooled to room temperature, filtered to remove the particle precipitate, then purified by a neutral alumina chromatography column, and column

chromatography was carried out with DCM as the eluent. The solution was concentrated to a viscous state, precipitated with methanol, and filtered to obtain the product. Finally, Soxhlet extraction was carried out with acetone, refluxed under heating and stirring at 90°C for 3 days. The product was vacuum dried to obtain a yellow powder solid. In this way, we prepared four polymers: PODPF-Cz, PDDPF-Cz, POBPF-Cz, and PDBPF-Cz.

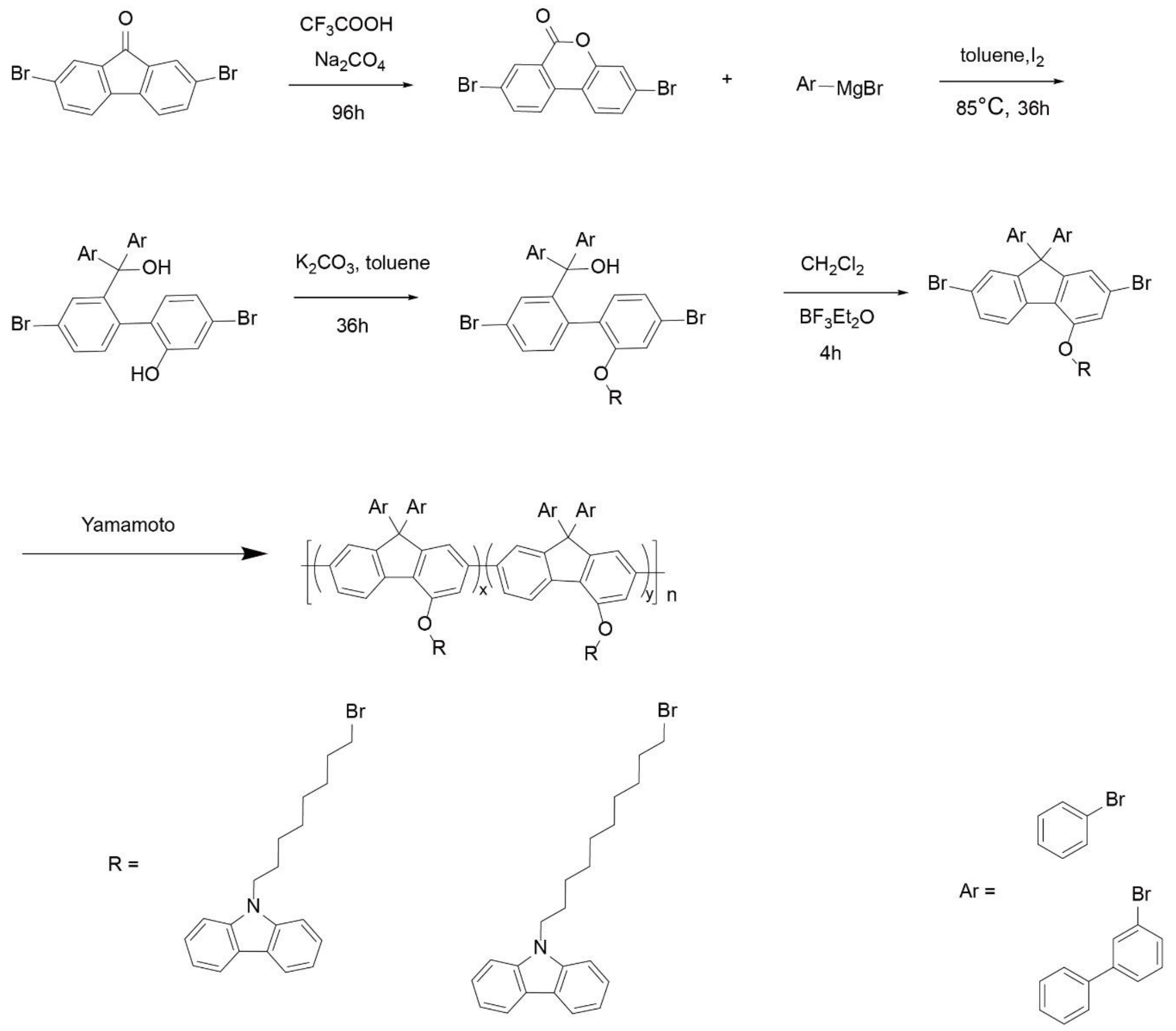

Scheme S1. The specific preparation steps of four polymers.

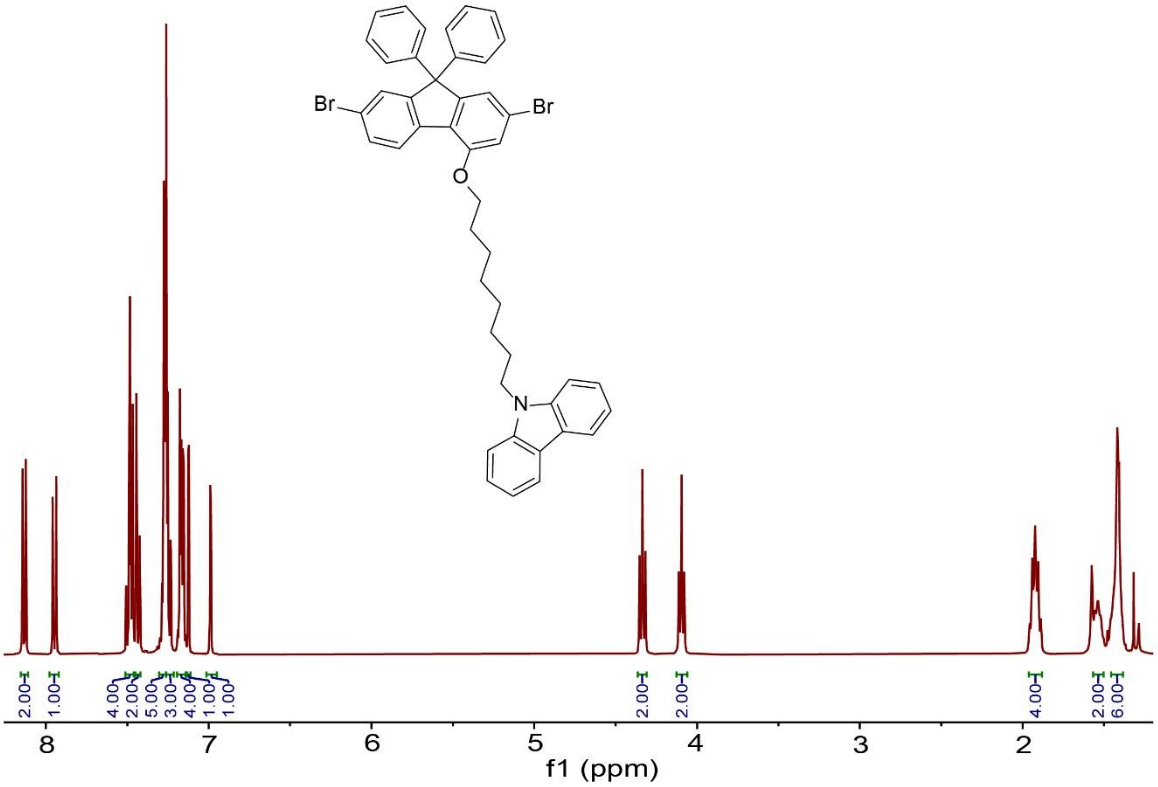
Br
Br
O
N
2.00
1.00
4.00
2.00
5.00
3.00
4.00
1.00
1.00
2.00
2.00
4.00
2.00
6.00
8 7 6 5 4 3 2
f1 (ppm)

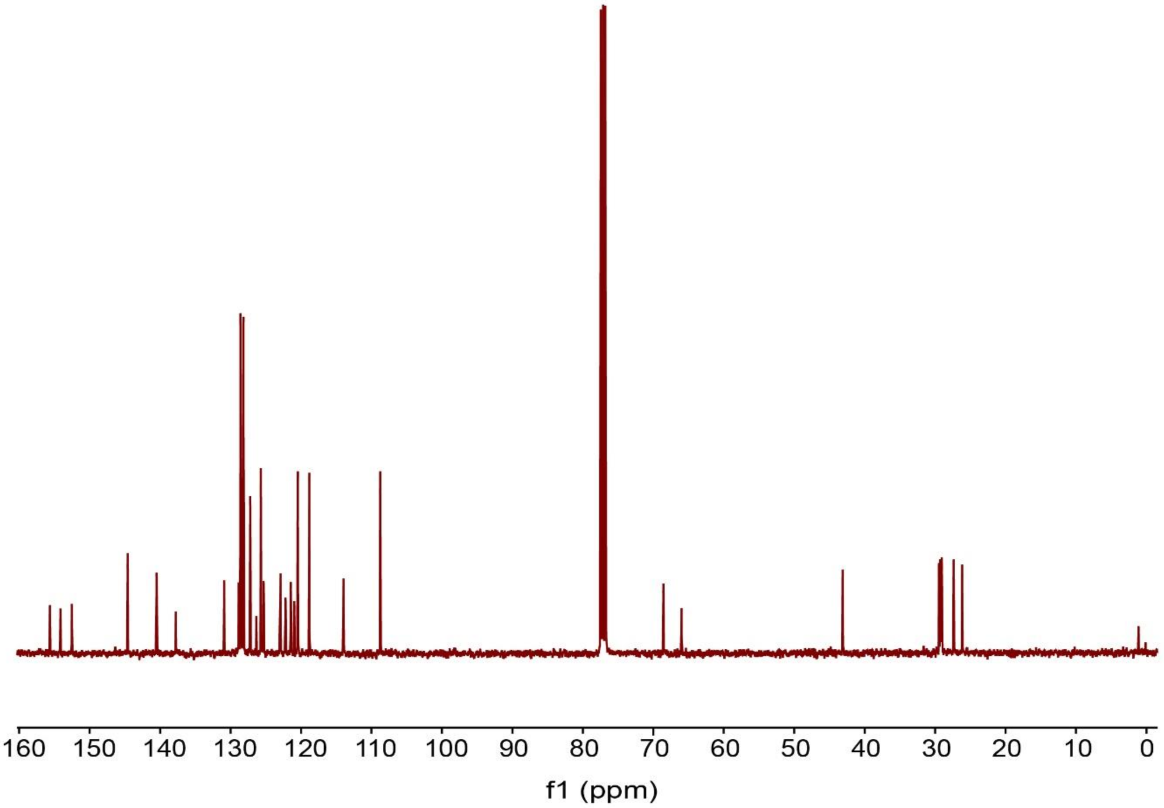
160 150 140 130 120 110 100 90 80 70 60 50 40 30 20 10 0
f1 (ppm)

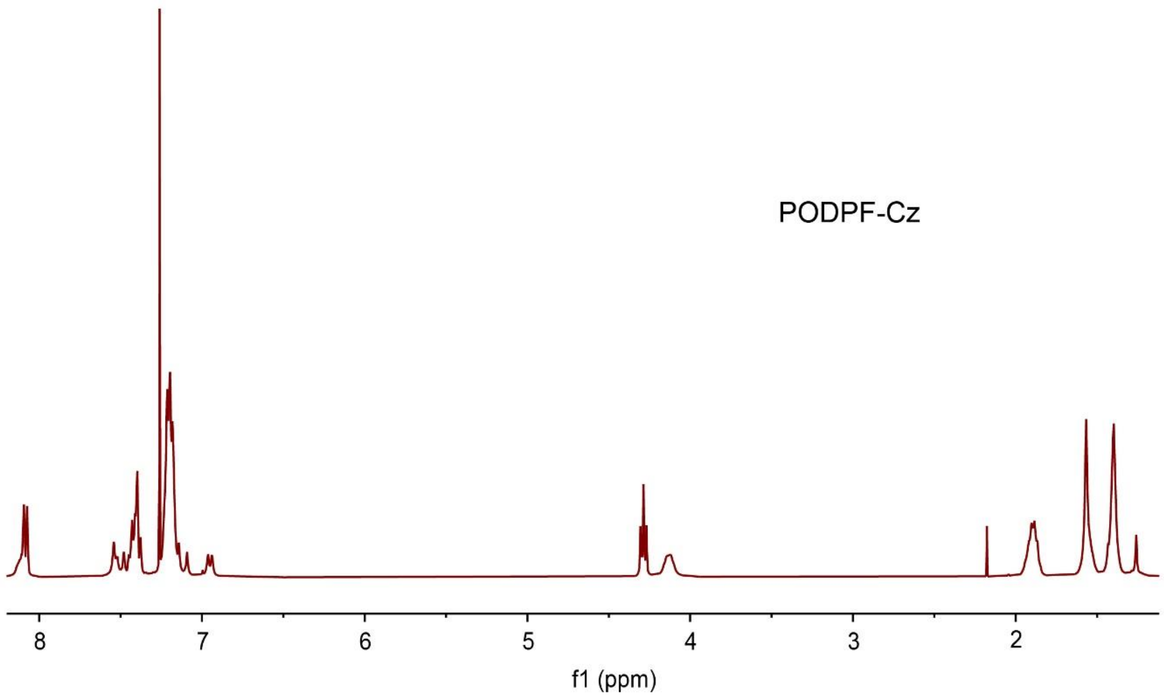

Figure S2. ^{1}H and ^{13}C NMR spectra of 9-(8-((2,7-dibromo-9,9-diphenyl-9H-fluoren-4-yl)oxy)octyl)-9H-carbazole (Monomer of PODPF-Cz) in $CDCl_3$. And ^{1}H NMR spectra of PODPF-Cz in $CDCl_3$.

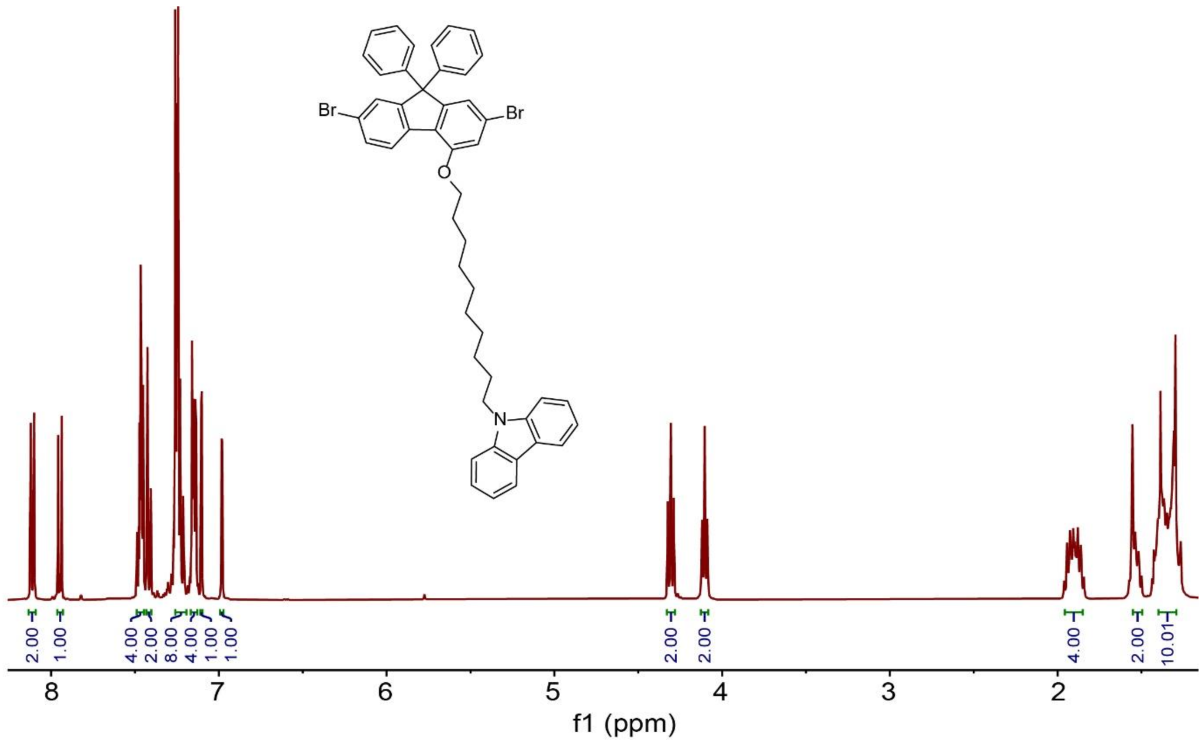

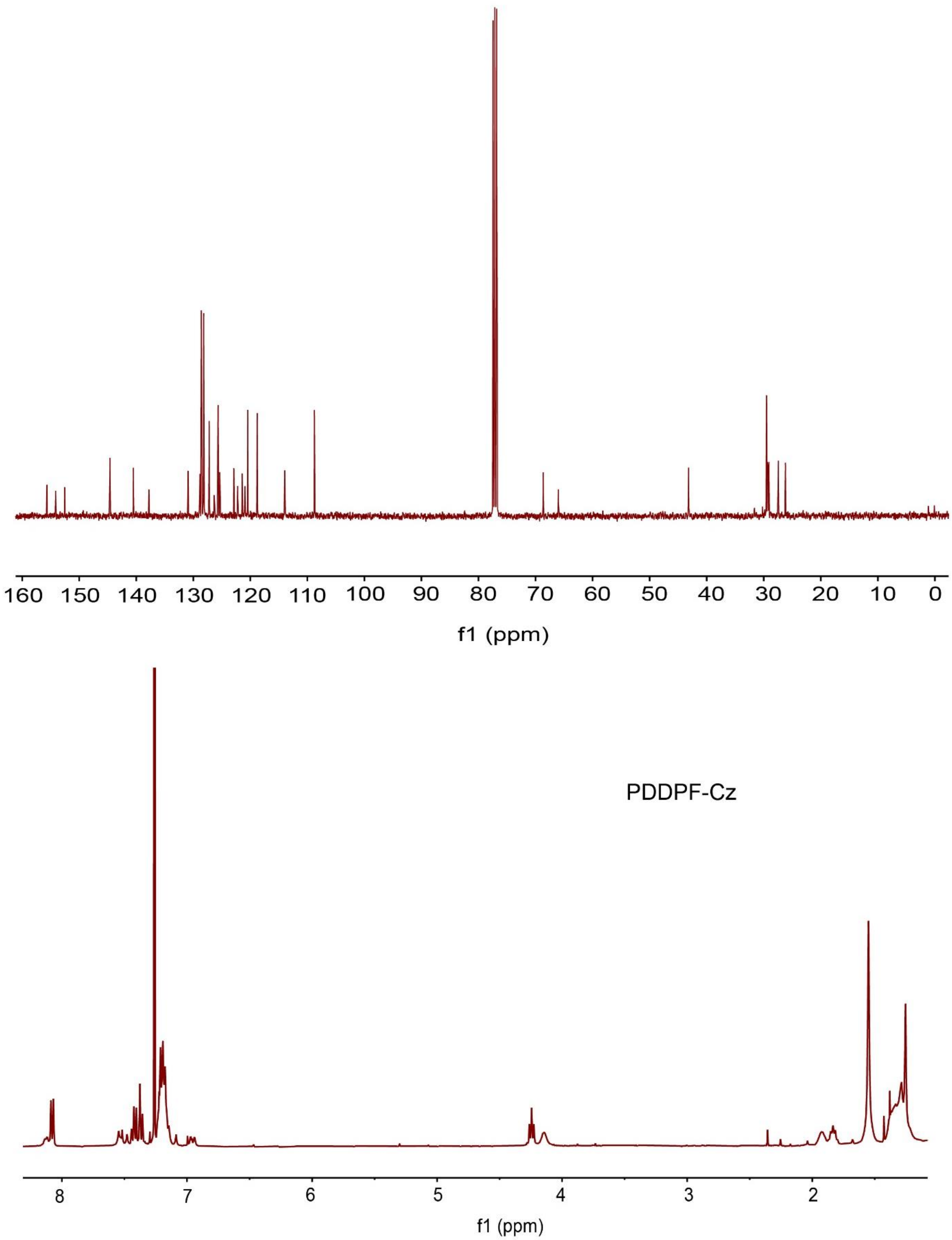

Figure S3. ^{1}H and ^{13}C NMR spectra of 9-(10-((2,7-dibromo-9,9-diphenyl-9H-fluoren-4-yl)oxy)decyl)-9H-carbazole (Monomer of PDDPF-Cz) in CDCl3. And 1H NMR spectra of PDDPF-Cz in CDC$_{l3}$.

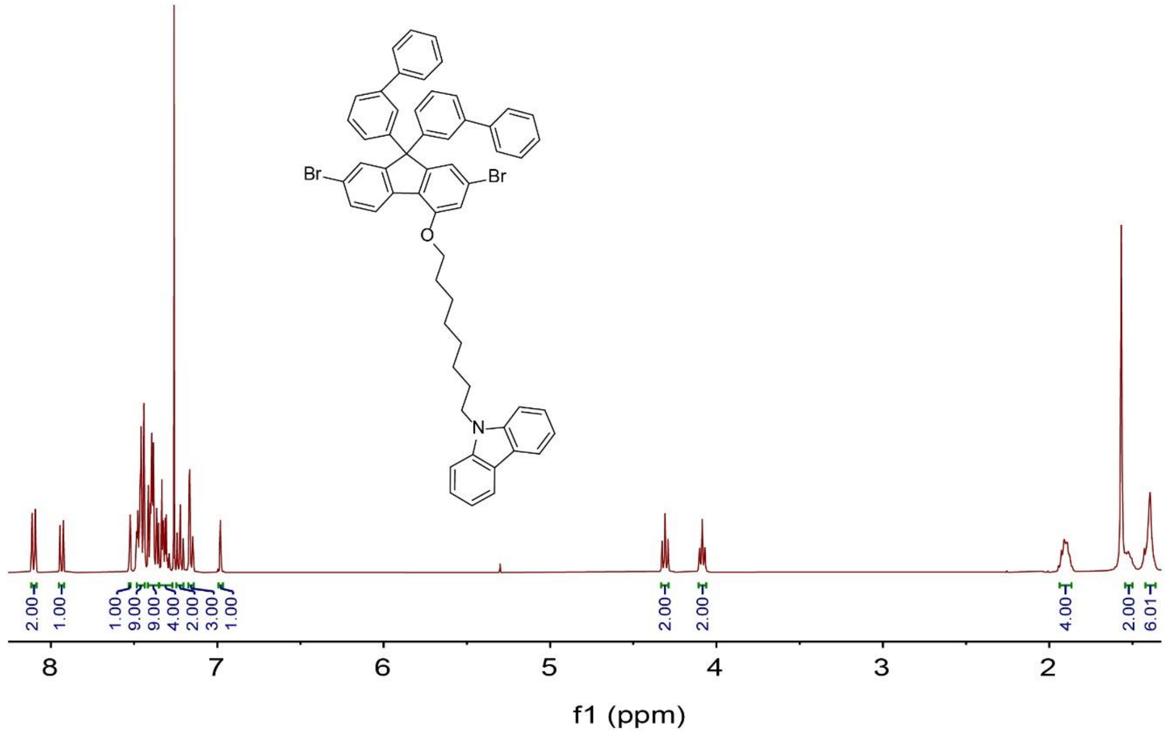
Br
Br
O
N
2.00
1.00
1.00
9.00
9.00
4.00
2.00
3.00
1.00
2.00
2.00
4.00
2.00
6.01
8 7 6 5 4 3 2
f1 (ppm)

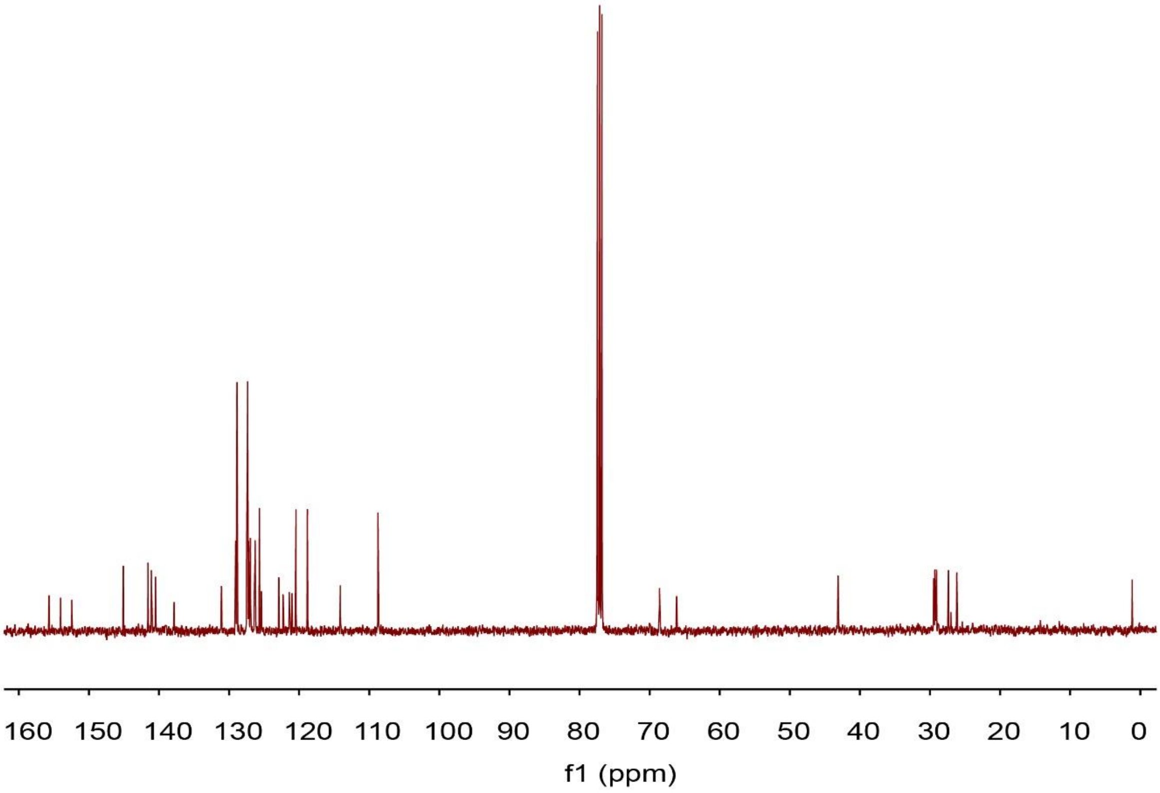
160 150 140 130 120 110 100 90 80 70 60 50 40 30 20 10 0
f1 (ppm)

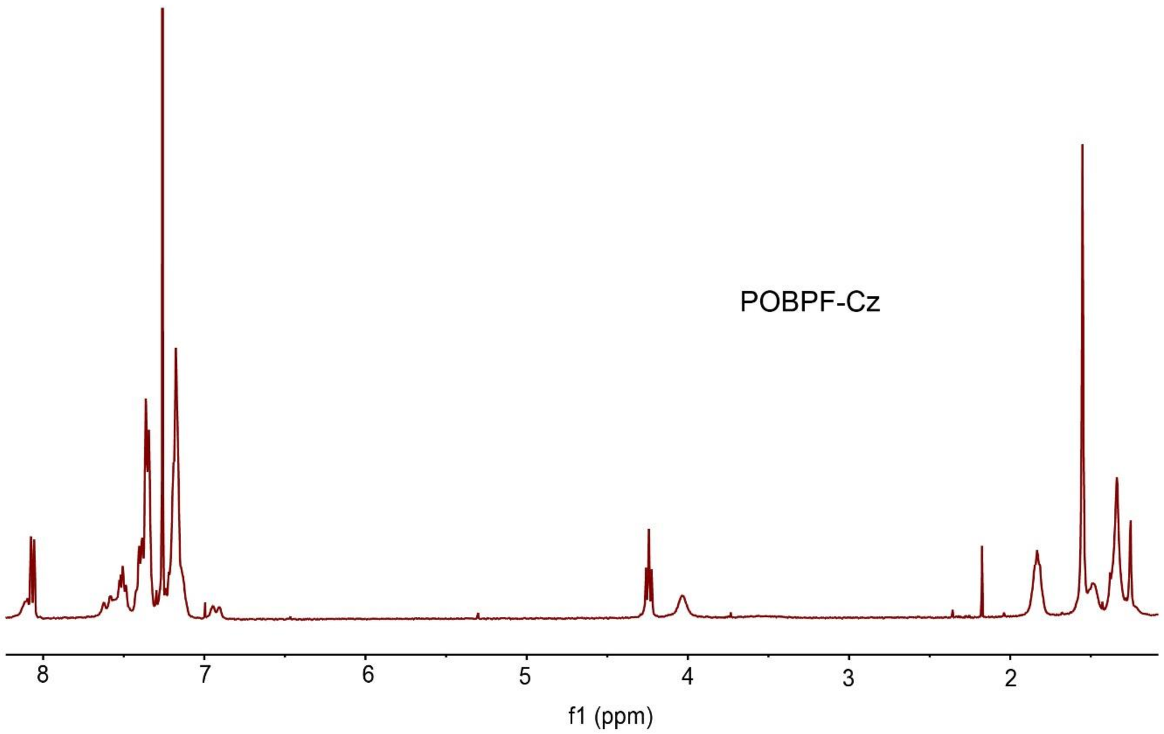

Figure S4. ^{1}H and ^{13}C NMR spectra of 9-(8-((9,9-di([1,1'-biphenyl]-3-yl)-2,7-dibromo-9H-fluoren-4-yl)oxy)octyl)-9H-carbazole (Monomer of POBPF-Cz) in $CDCl_3$. And ^{1}H NMR spectra of POBPF-Cz in $CDCl_3$.

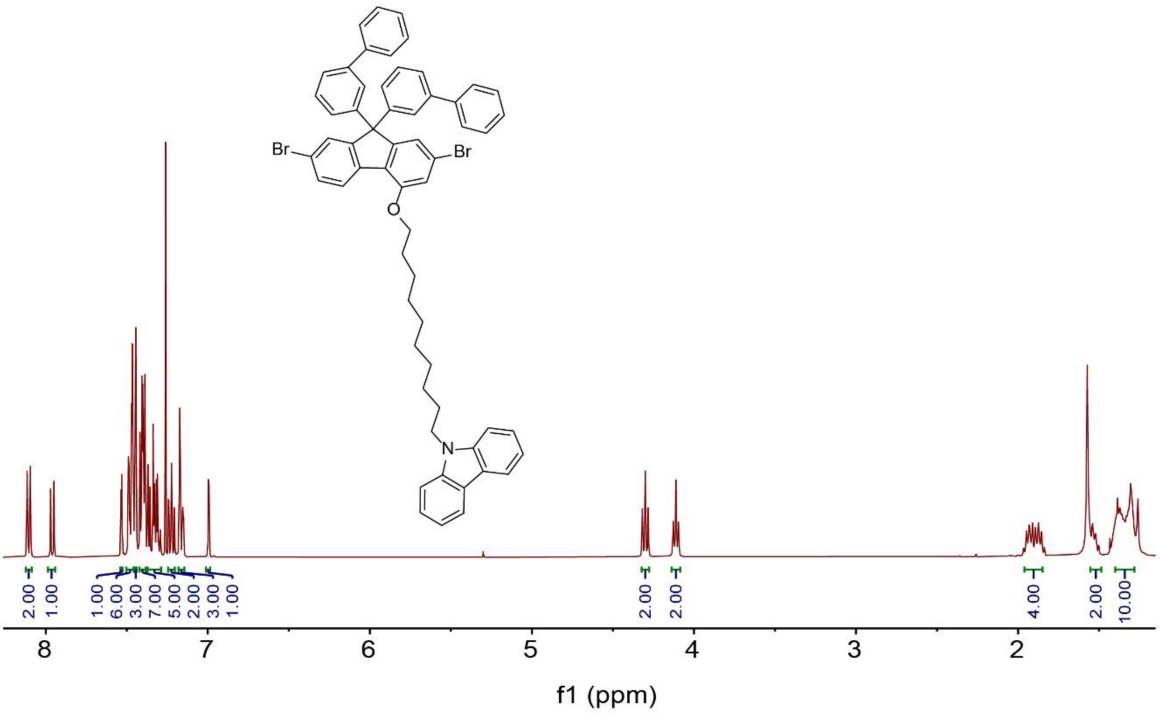

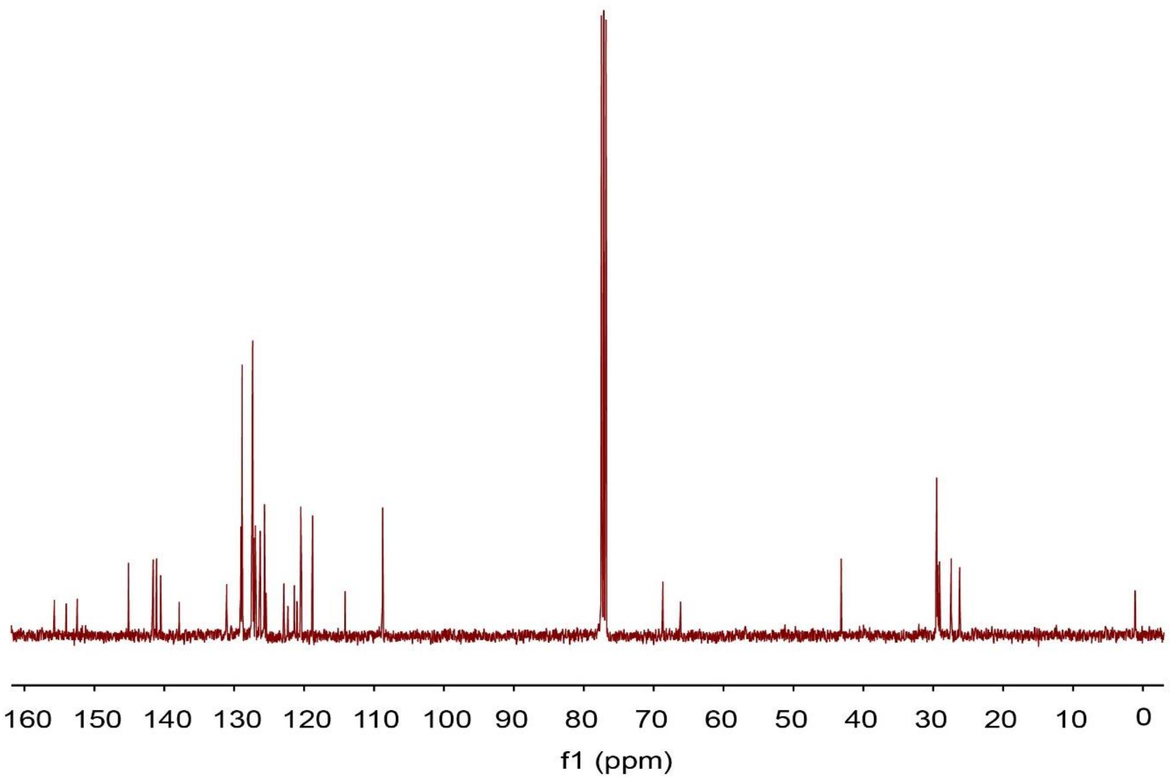

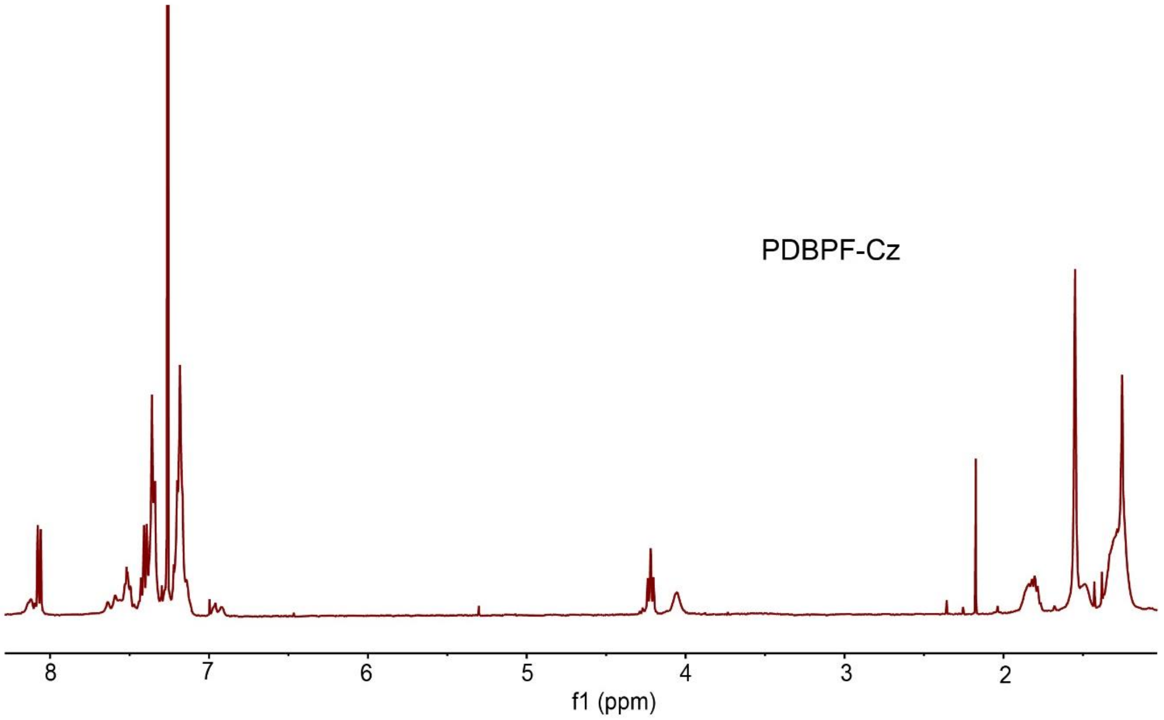

Figure S5. ^{1}H and ^{13}C NMR spectra of 9-(10-((9,9-di([1,1'-biphenyl]-3-yl)-2,7-dibromo-9H-fluoren-4-yl)oxy)decyl)-9H-carbazole (Monomer of PDBPF-Cz) in $CDCl_3$. And ^{1}H NMR spectra of PDBPF-Cz in $CDCl_3$.

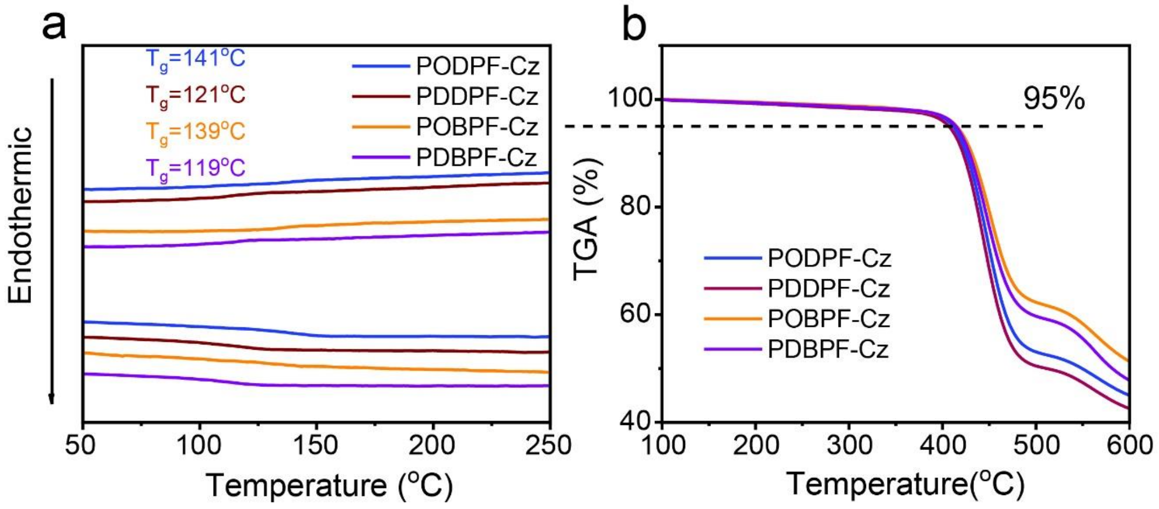

Figure S6. (a) TGA and (b) DSC curves of PODPF-Cz, PDDPF-Cz, POBPF-Cz and PDBPF-Cz. Heating rate was 10 K/min under nitrogen atmosphere.

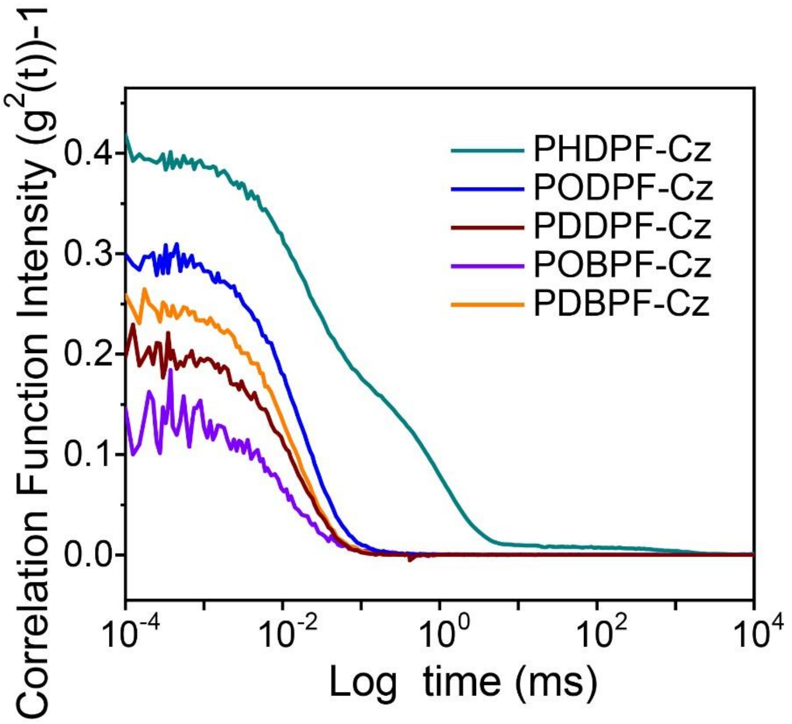

Figure S7. DLS curves of PHDPF-Cz, PODPF-Cz, PDDPF-Cz, POBPF-Cz and PDBPF-Cz toluene solutions (1 mg/mL).

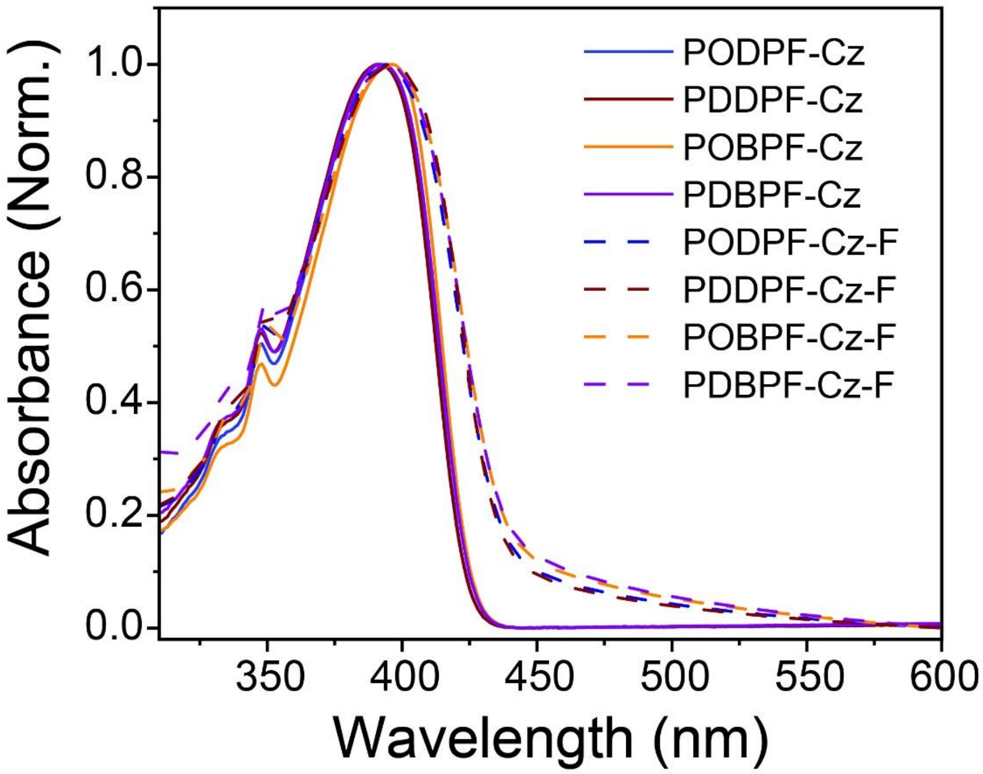

Figure S8. Absorbance spectra of PODPF-Cz, PDDPF-Cz, POBPF-Cz and PDBPF-Cz in solutions and solid films.

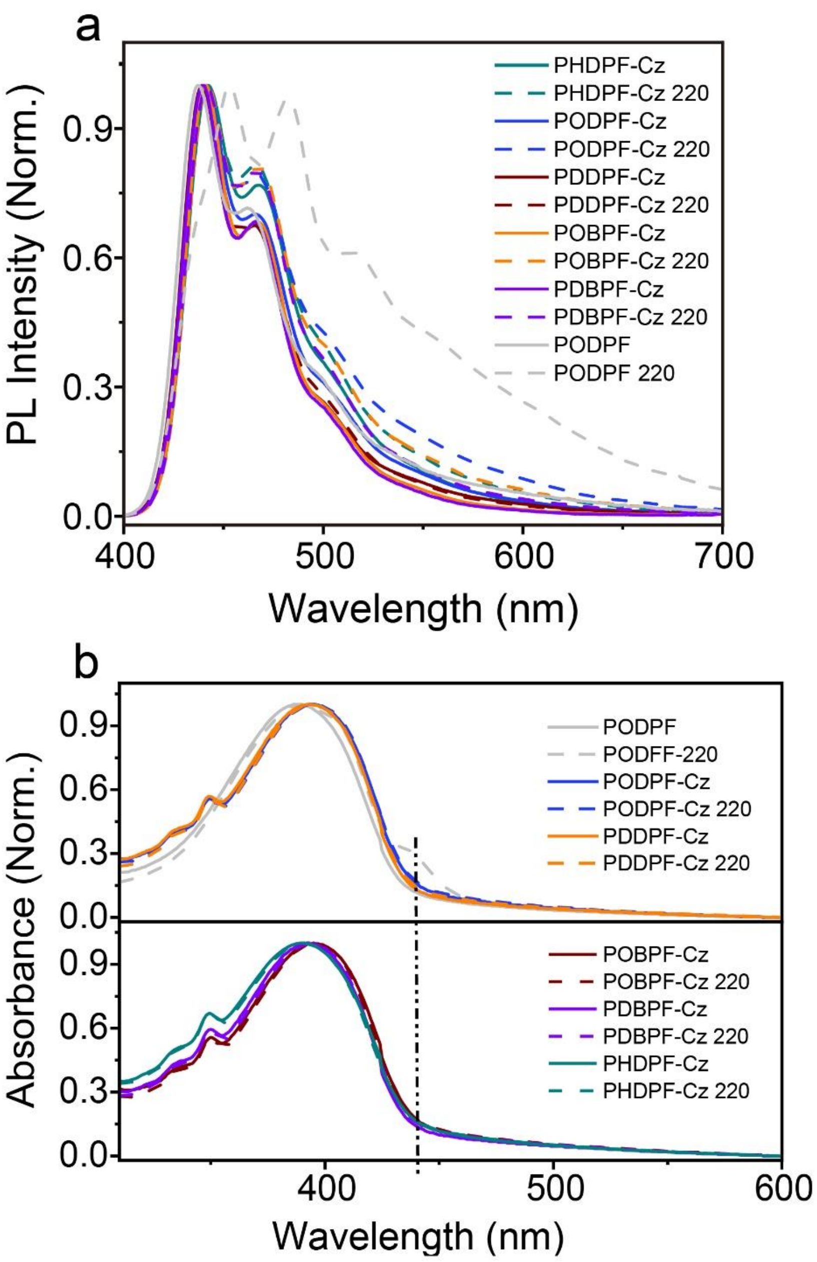

Figure S9. (a) Absorbance and (b) PL spectra of PHDPF-Cz, PODPF-Cz, PDDPF-Cz, POBPF-Cz, PDBPF-Cz and PODPF films coated from toluene solutions under 220 ^{o}C in the N_2 atmosphere.

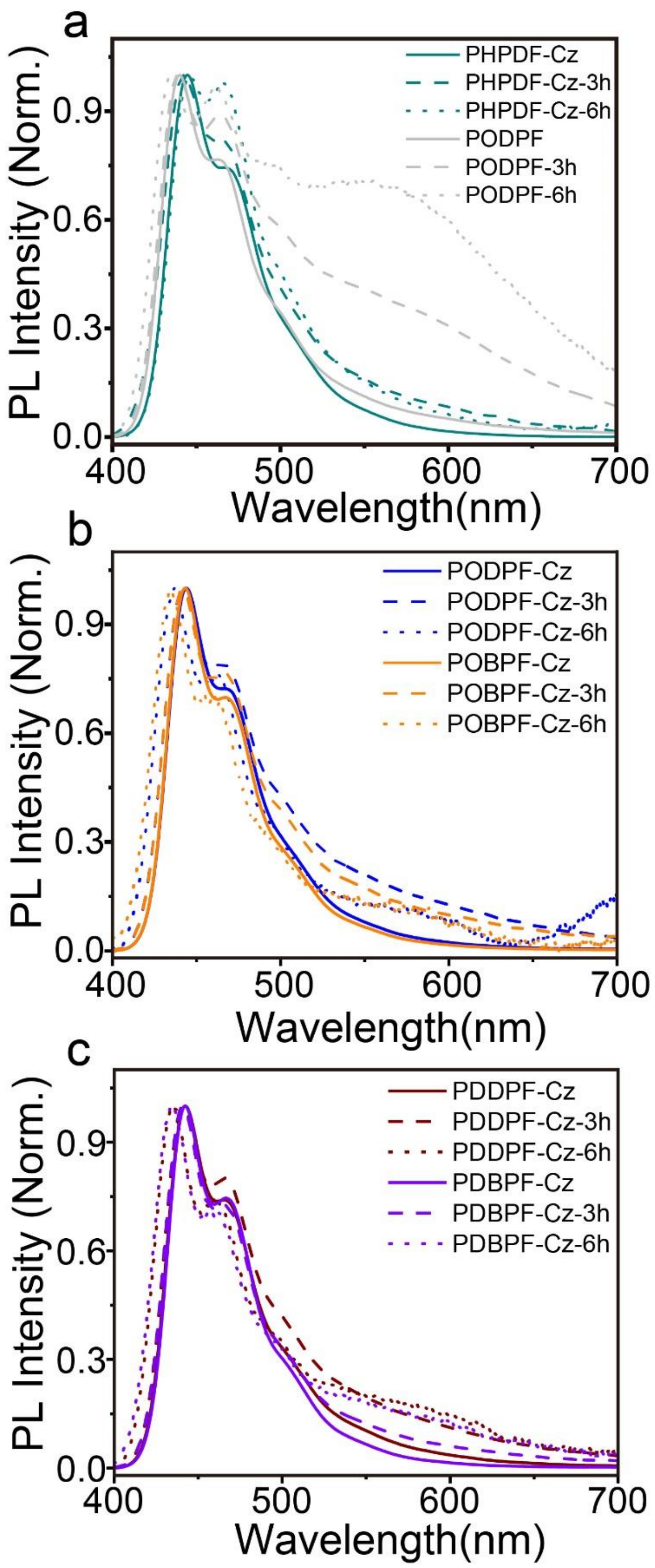

Figure S10. PL spectra of PHDPF-Cz, PODPF-Cz, POBPF-Cz, PDDPF-Cz and PDBPF-Cz coated film from toluene solutions with a concentration of 10 mg/ml aged for 3h and 6h. Aged films were prepared by keeping the films under a UV lamp in an ambient atmosphere several times.

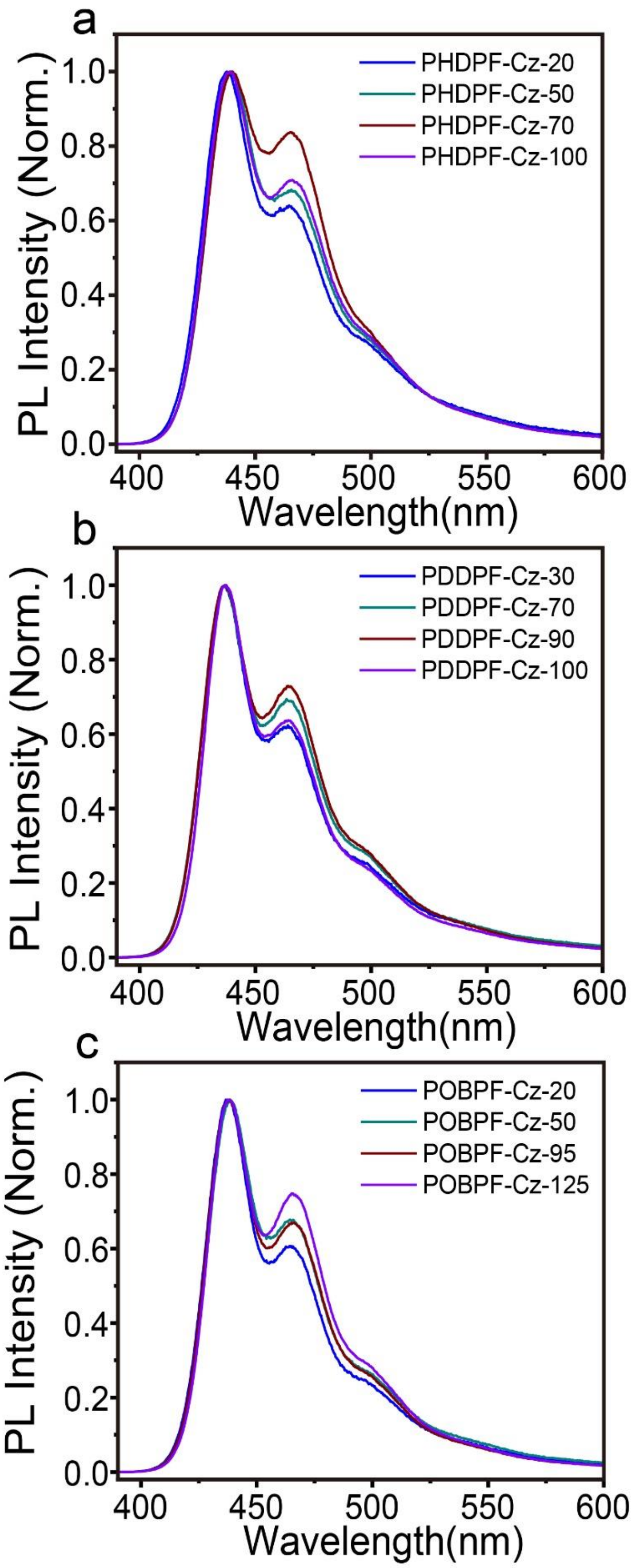

Figure S11. PL spectra of PHDPF-Cz, PDDPF-Cz and POBPF-Cz films coated from toluene solutions with the different thickness. The thickness of polymer films can be tuned via controlling the concentration of 5 mg/ml, 10 mg/ml, 15 mg/ml and 20 mg/ml.

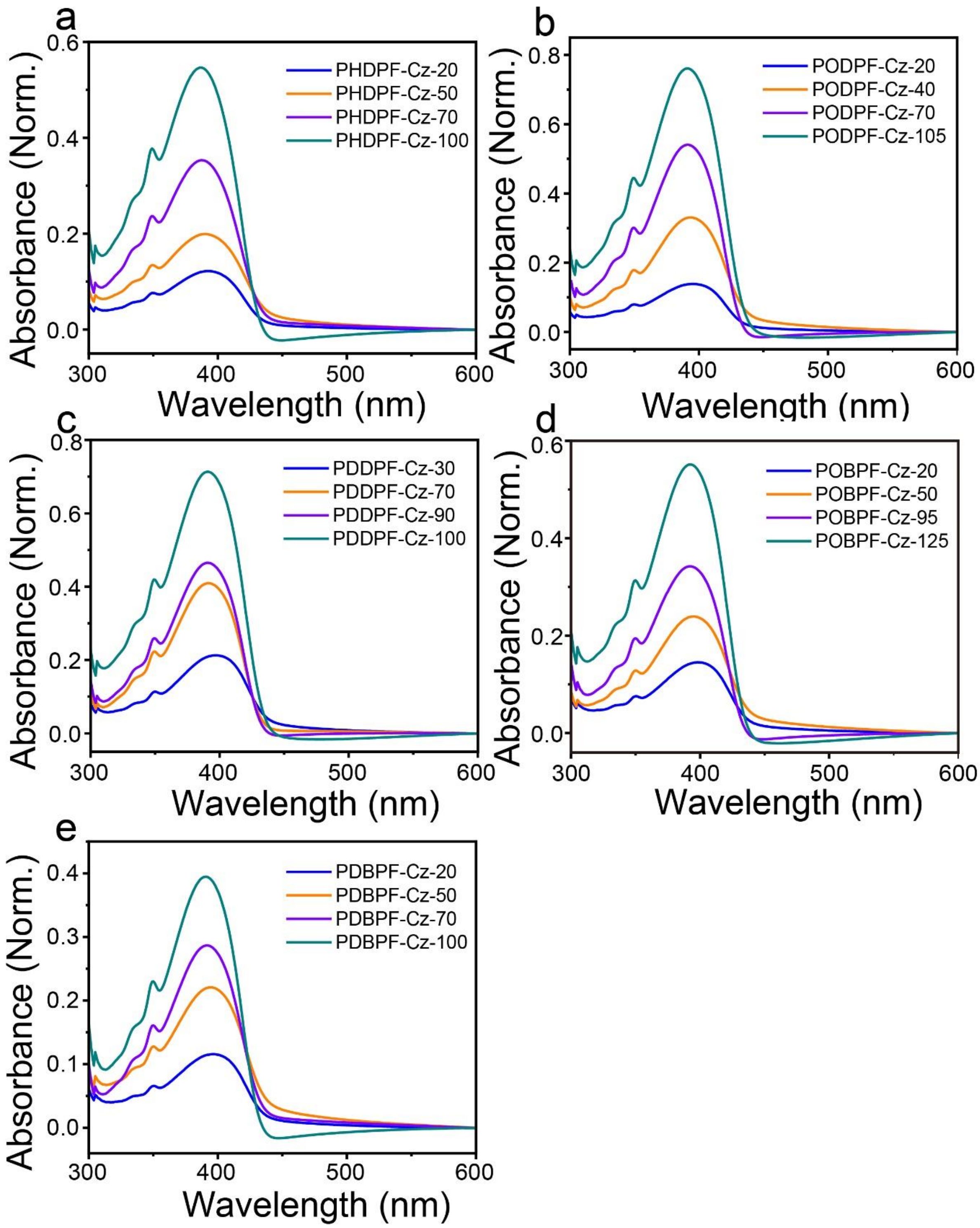

Figure S12. Absorbance spectra of PHDPF-Cz, PODPF-Cz, PDDPF-Cz, POBPF-Cz and PDBPF-Cz films coated from toluene solutions with the different thickness. The thickness of polymer films can be tuned via controlling the concentration of 5 mg/ml, 10 mg/ml, 15 mg/ml and 20 mg/ml.

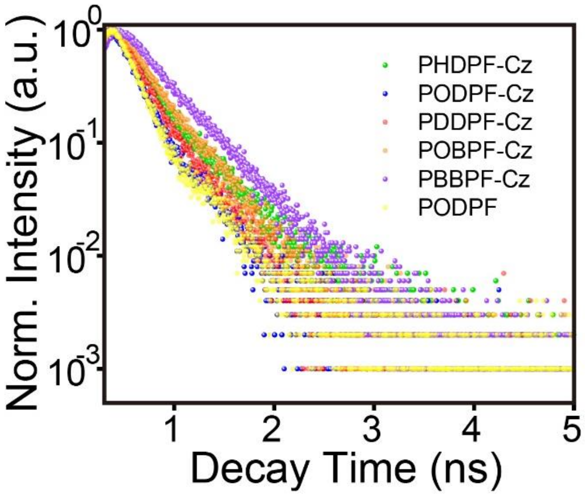

PHDPF-Cz	0.378
PODPF-Cz	0.272
POBPF-Cz	0.364
PDDPF-Cz	0.313
PDBPF-Cz	0.446
PODPF	0.296

Figure S13. Fluorescence lifetime of PHDPF-Cz, PODPF-Cz, PDDPF-Cz, POBPF-Cz, PDBPF-Cz and PODPF films coated from toluene solutions.

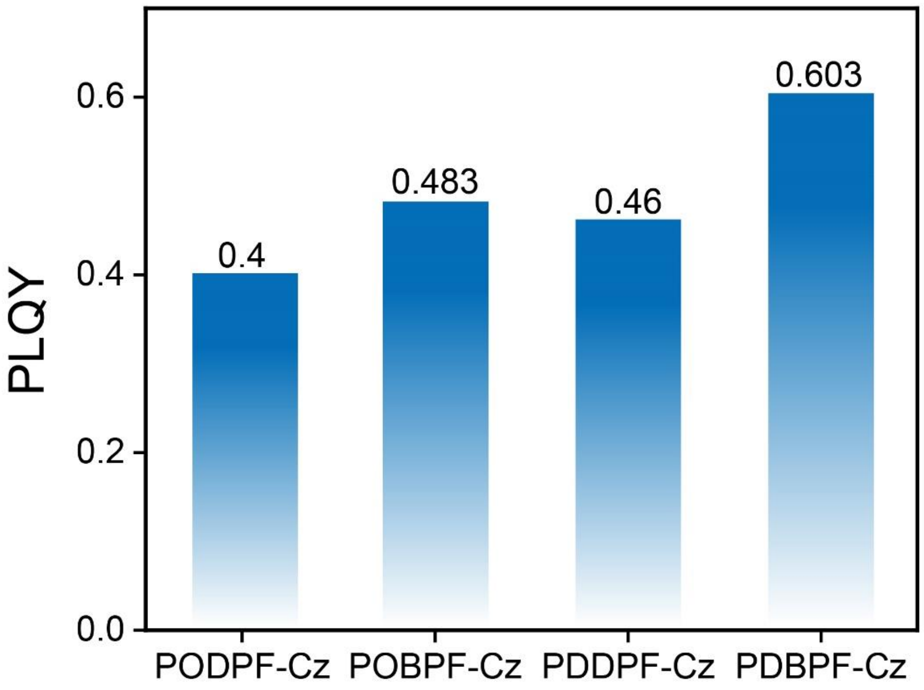

Figure S14. PLQY of PODPF-Cz, POBPF-Cz, PDDPF-Cz and PDBPF-Cz films coated from toluene solutions.

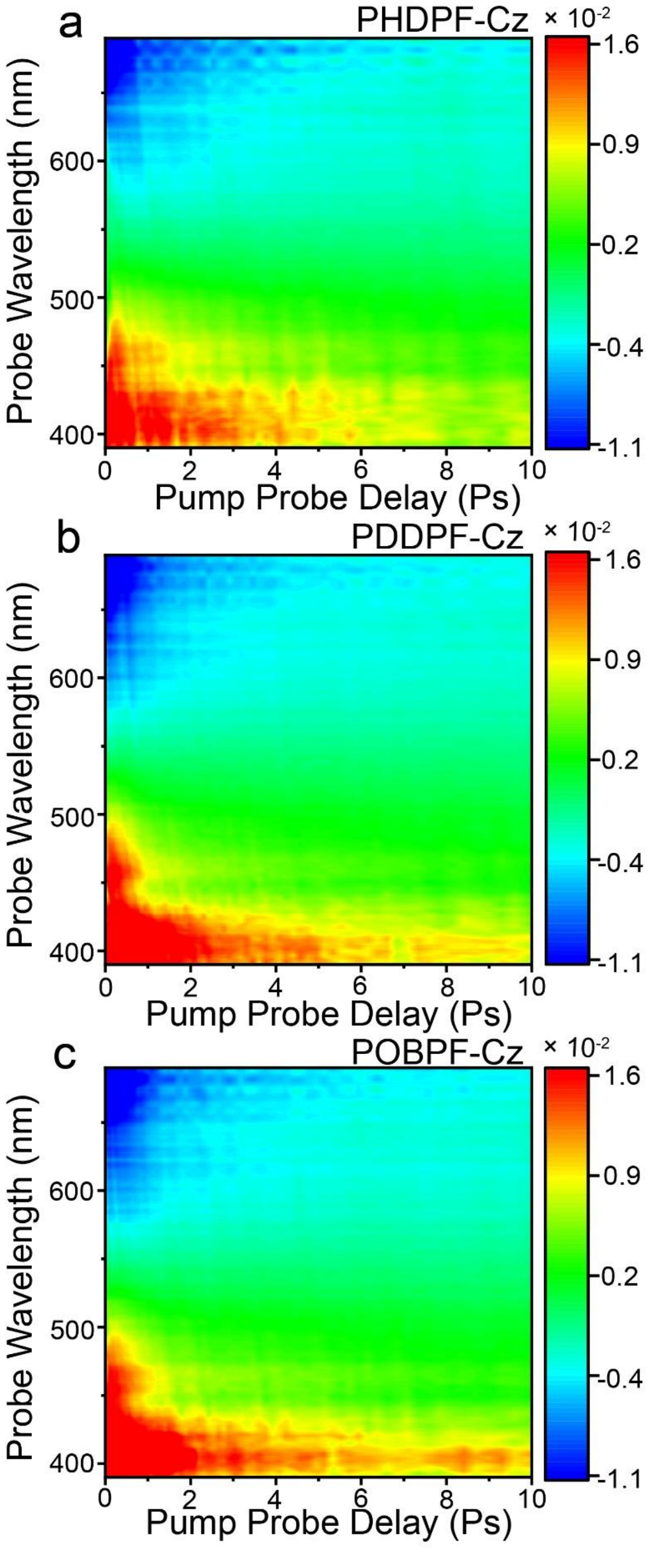

Figure S15. TA contour plots of PHDPF-Cz, PDDPF-Cz and POBPF-Cz solid films.

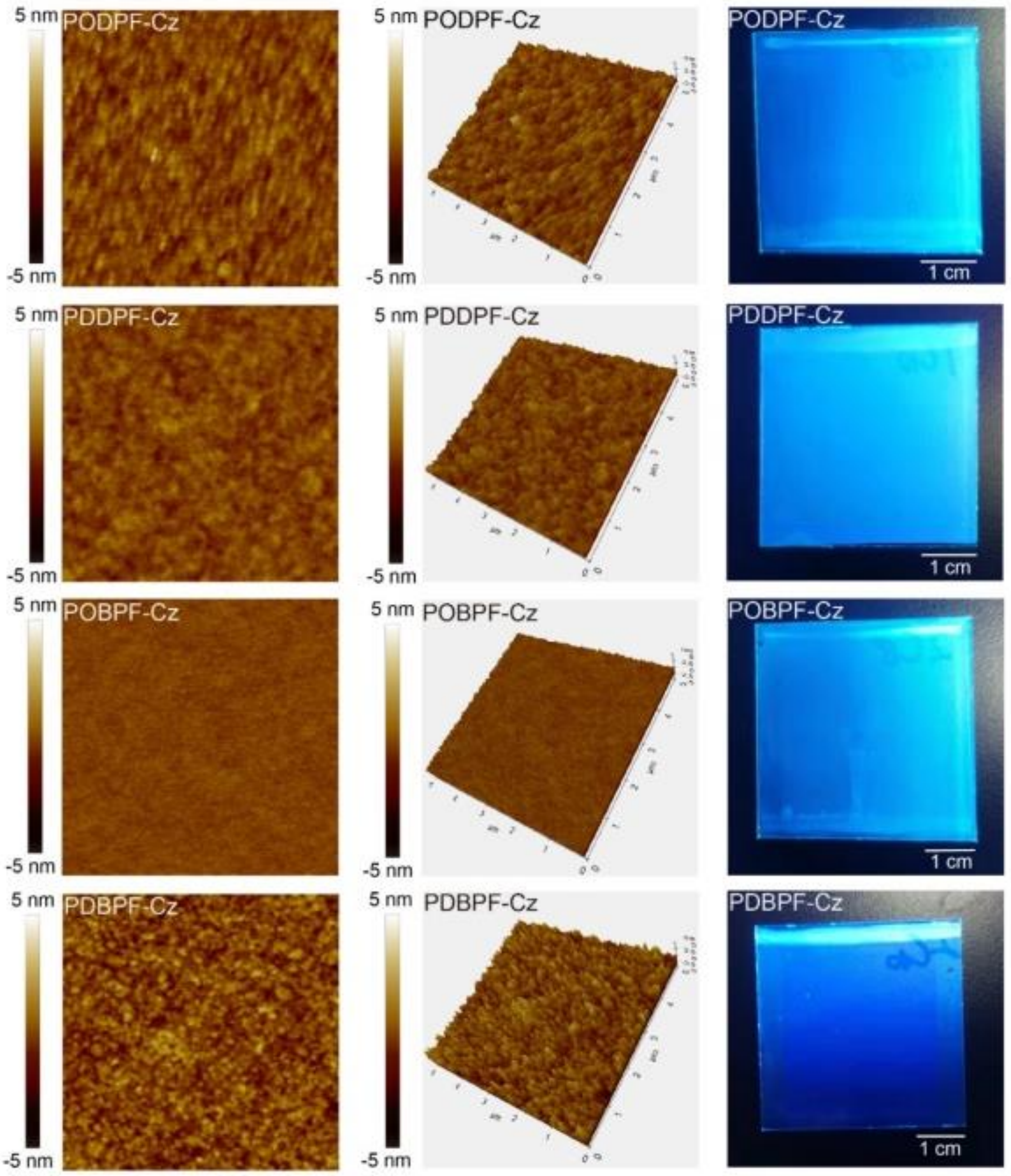

Figure S16. AFM images of large-area blade-coated film from various polymers, together with their photograph under UV lamp (365 nm).

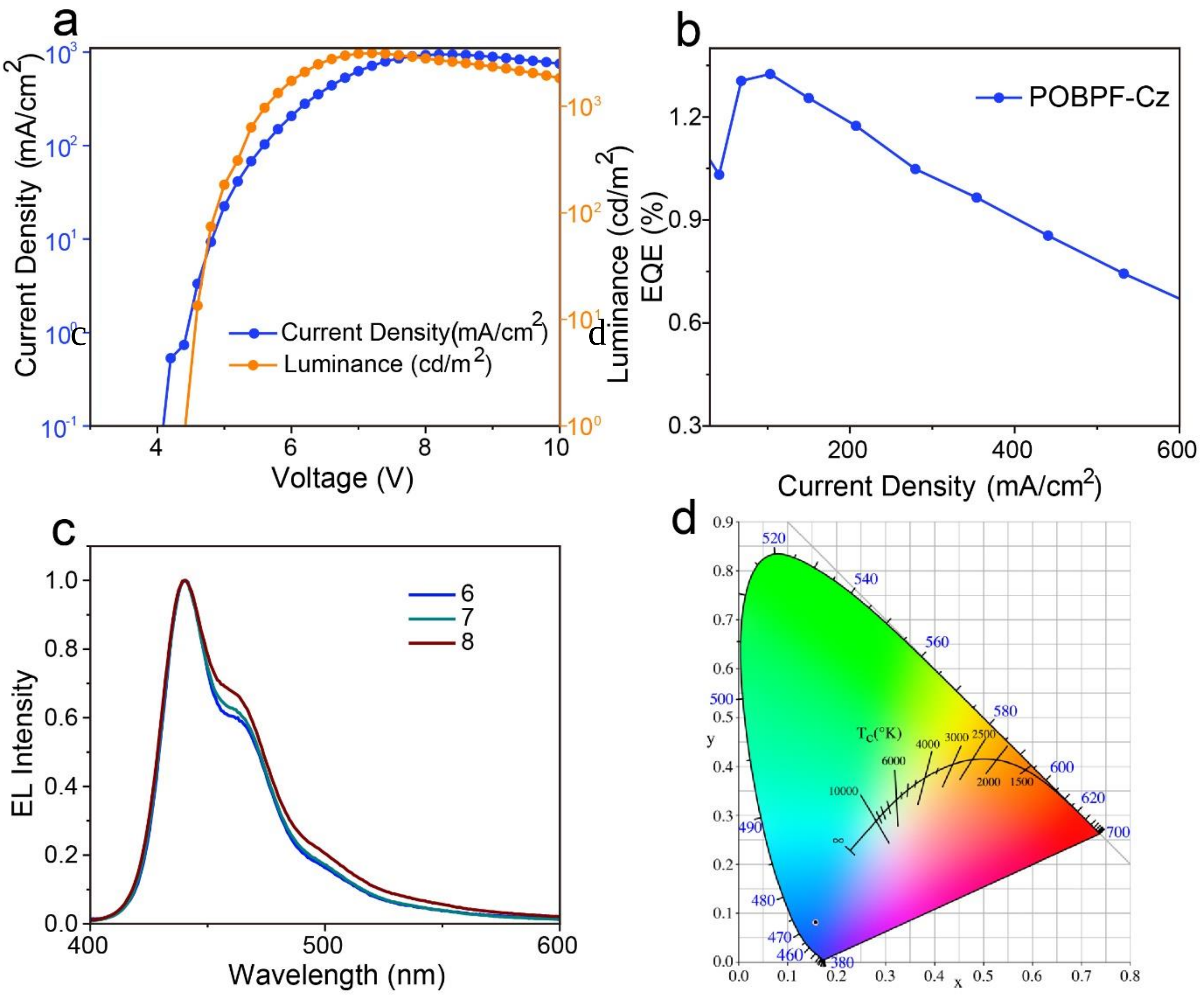

Figure S17. **(a)** Current density–luminance–voltage characteristics of the POBPF-Cz spin-coated devices. **(b)** external quantum efficiencies (EQE) of the POBPF-Cz spin-coated devices. **(c)** EL spectra of the POBPF-Cz spin-coated devices with different voltage. **(d)** CIE spectra of the POBPF-Cz spin-coating devices.

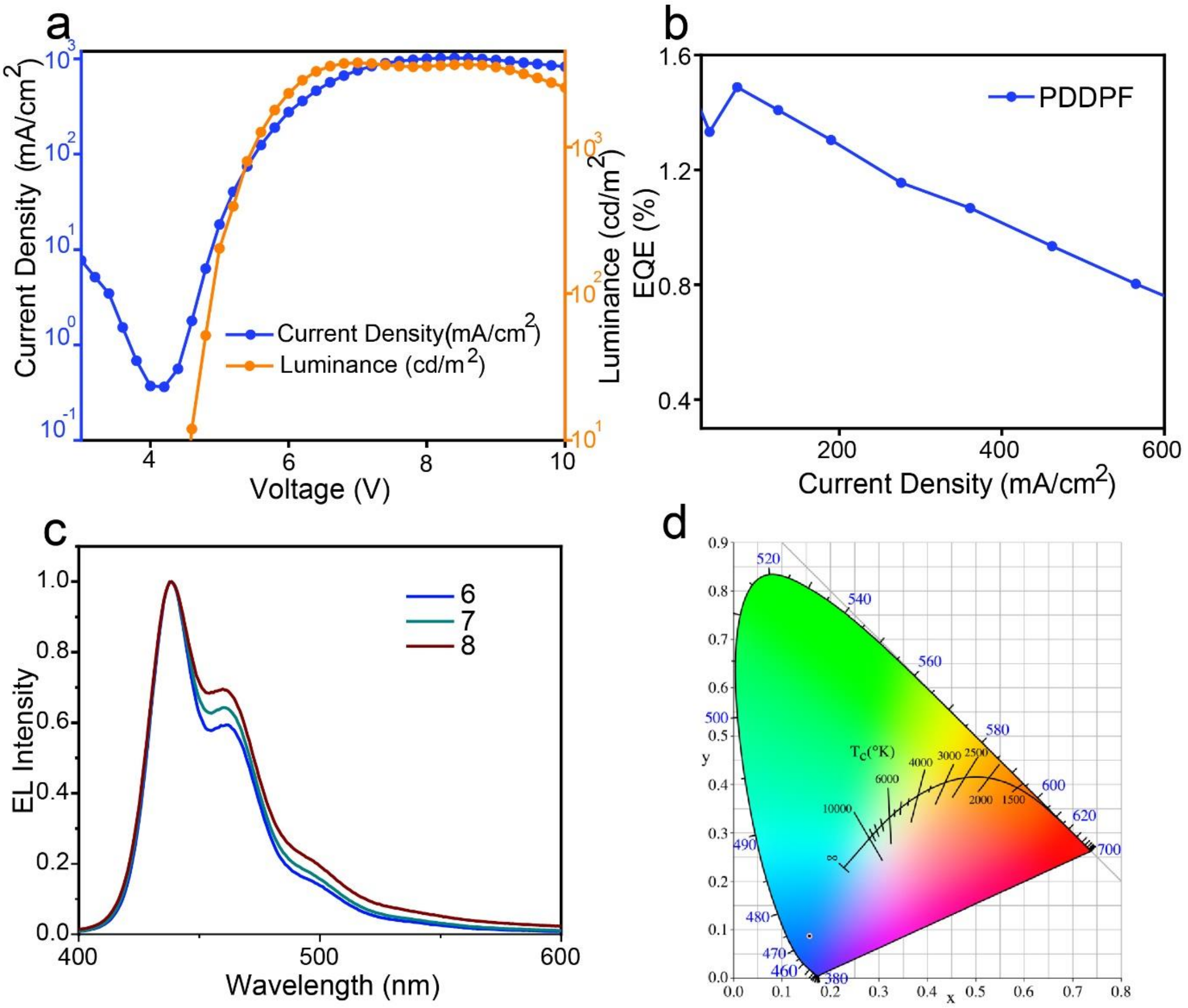

Figure S18. **(a)** Current density–luminance–voltage characteristics of the PDDPF-Cz spin-coated devices. **(b)** external quantum efficiencies (EQE) of the PDDPF-Cz spin-coated devices. **(c)** EL spectra of the PDDPF-Cz spin-coated devices with different voltage. **(d)** CIE spectra of the PDDPF-Cz spin-coating devices.

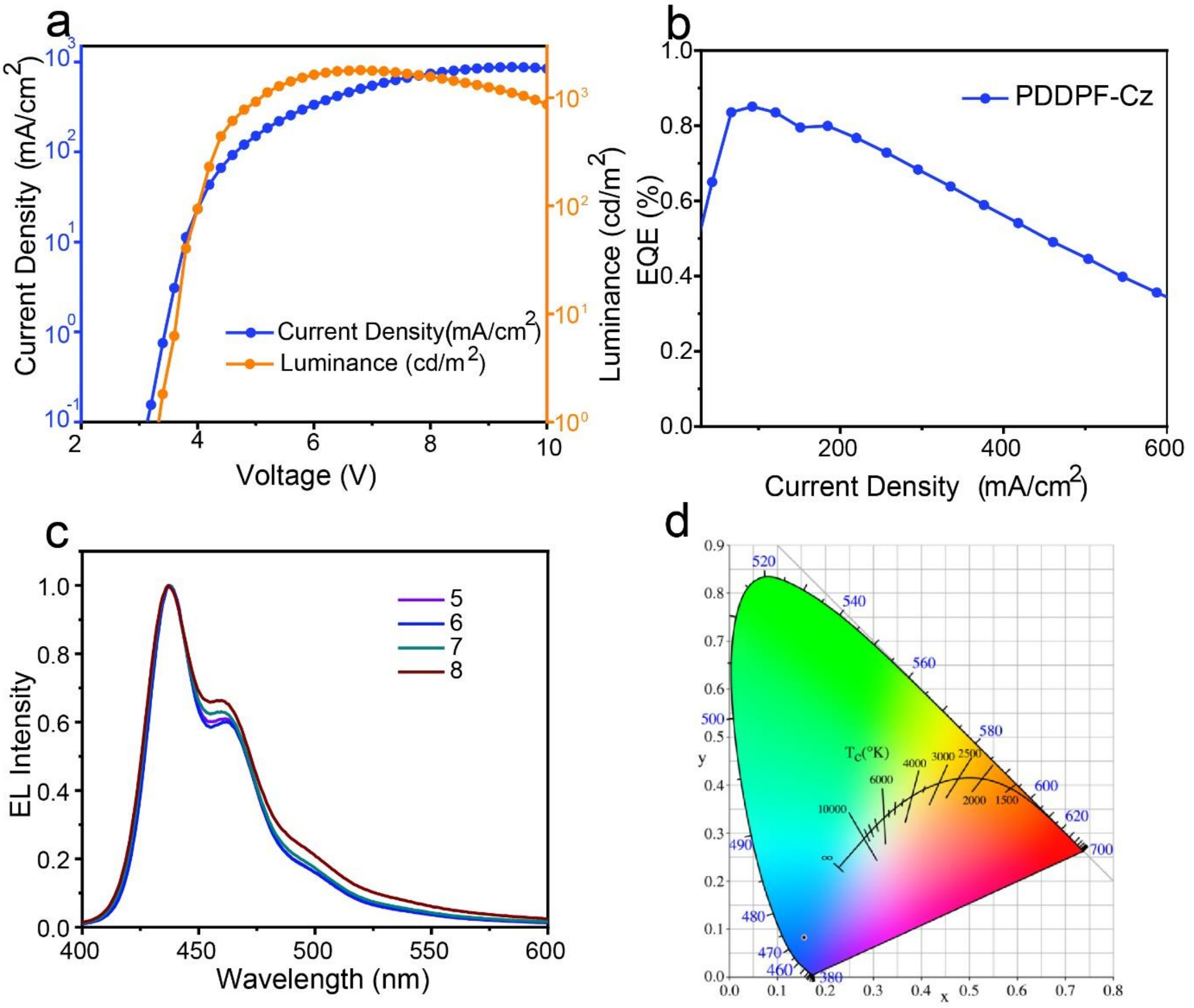

Figure S19. **(a)** Current density–luminance–voltage characteristics of the POBPF-Cz blade-coated devices. **(b)** external quantum efficiencies (EQE) of the POBPF-Cz blade-coated devices. **(c)** EL spectra of the POBPF-Cz blade-coated devices with different voltage. **(d)** CIE spectra of the PO9BPF-Cz blade-coating devices.

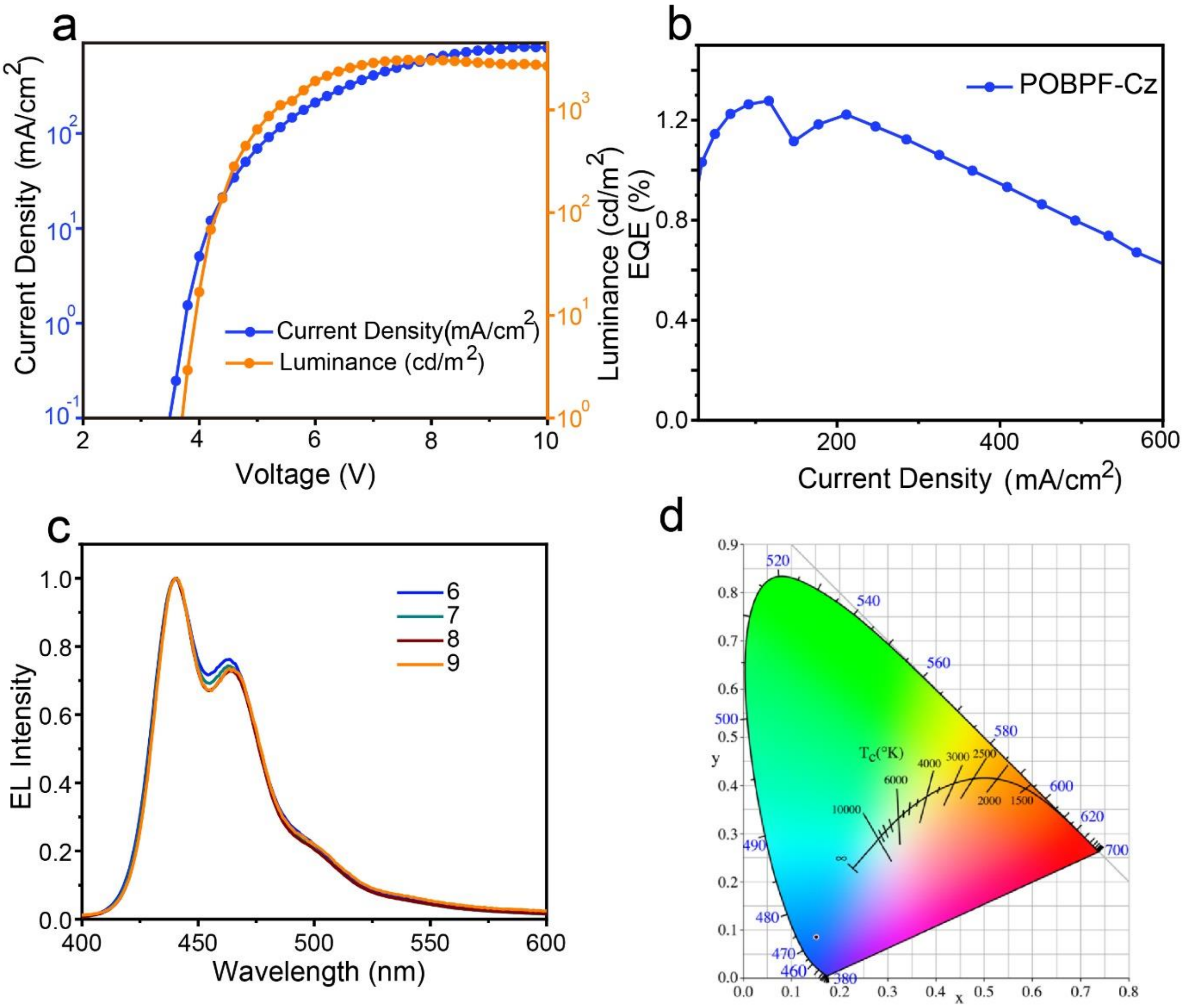

Figure S20. **(a)** Current density–luminance–voltage characteristics of the PDDPF-Cz blade-coated devices. **(b)** external quantum efficiencies (EQE) of the PDDPF-Cz blade-coated devices. **(c)** EL spectra of the PDDPF-Cz blade-coated devices with different voltage. **(d)** CIE spectra of the PDDPF-Cz blade-coating devices.

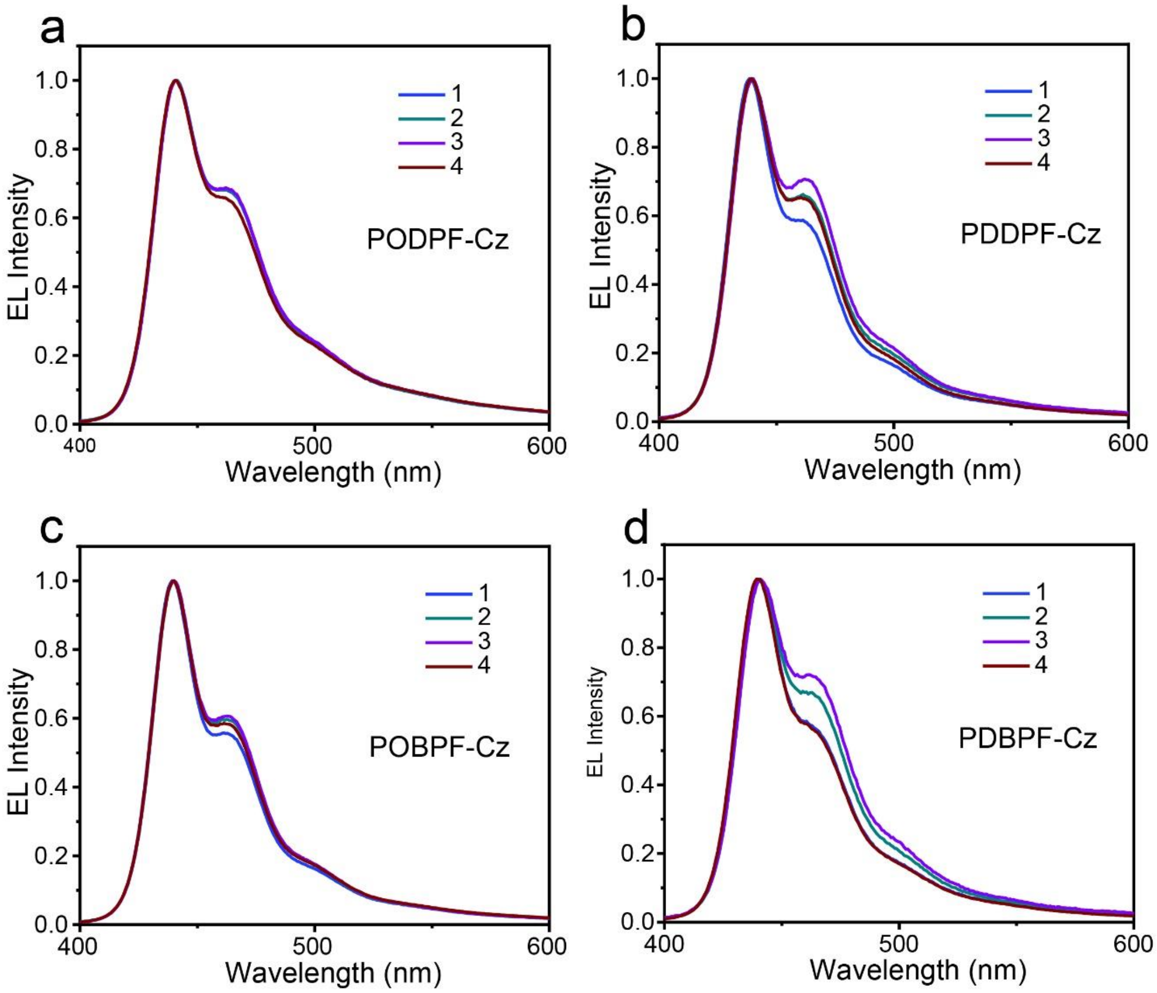

Figure S21. EL spectra of the PODPF-Cz, PDDPF-Cz, POBPF-Cz and PDBPF-Cz spin-coated devices with different positions of the same film under the same voltage.

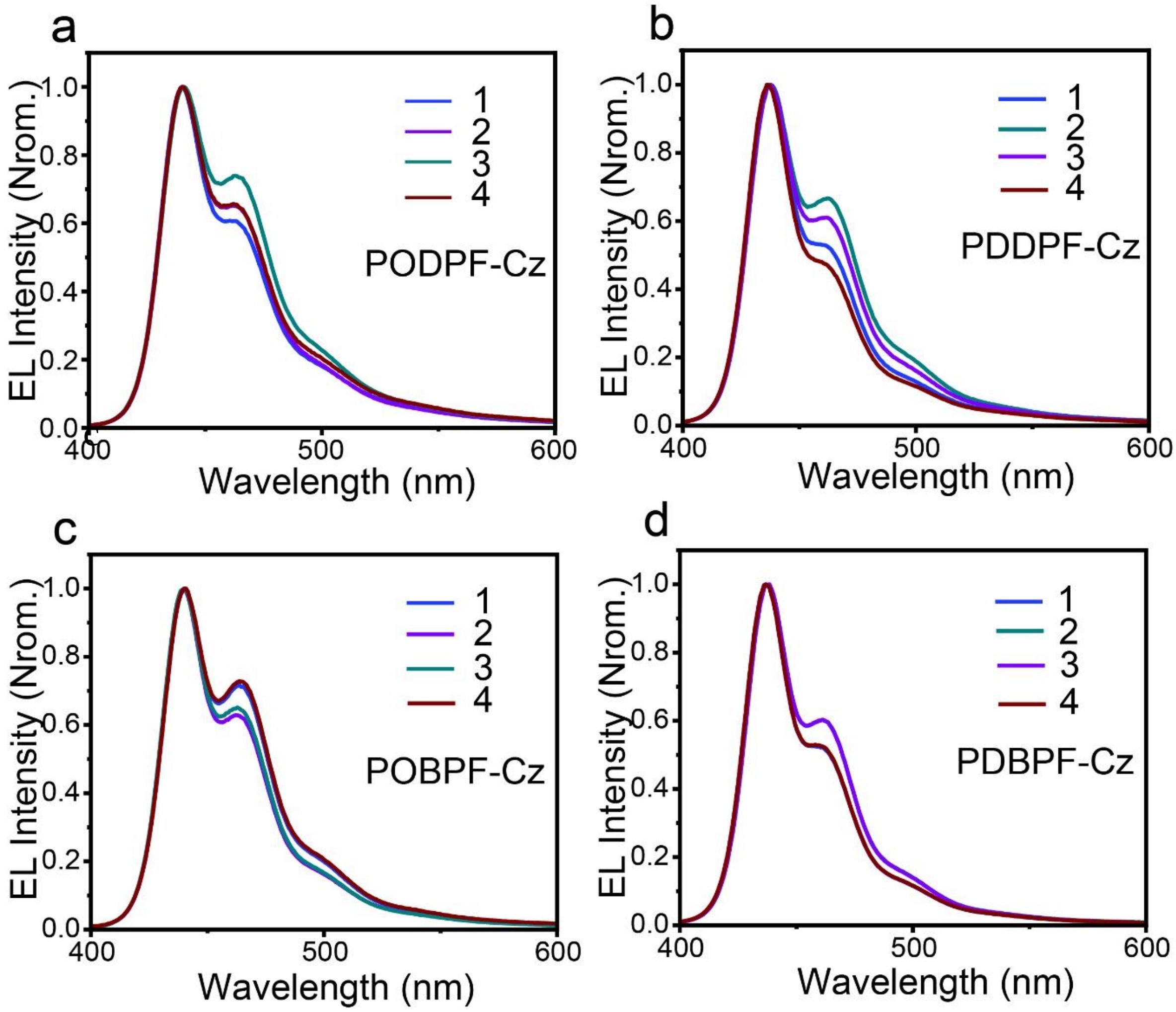

Figure S22. EL spectra of the PODPF-Cz, PDDPF-Cz, POBPF-Cz and PDBPF-Cz blade-coated devices with different positions of the same film under the same voltage.

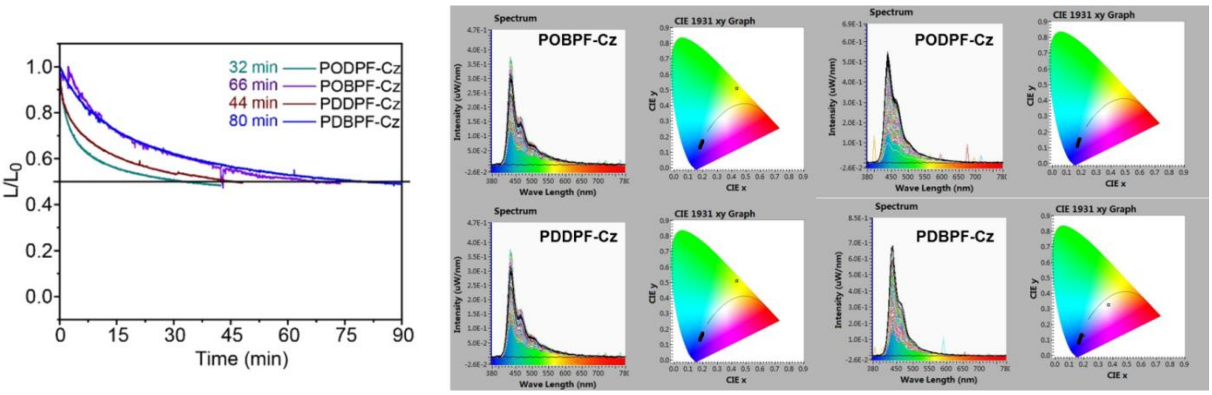

Figure S23. Half-life time (L/L_0) of large-area deep-blue blade-coated PLEDs based on our novel materials, together with the current-density dependent CIE (color purity of deep-blue emission).

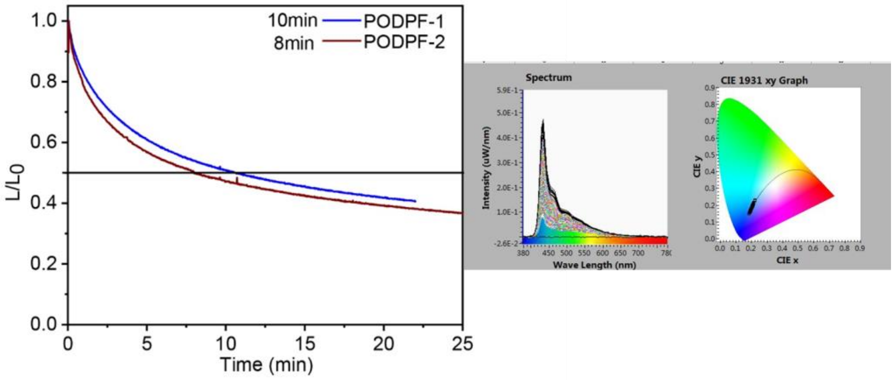

Figure S24. Half-life time (L/L_0) of large-area deep-blue blade-coated PLEDs based on controlled PODPF, together with the current-density dependent CIE (color purity of deep-blue emission).

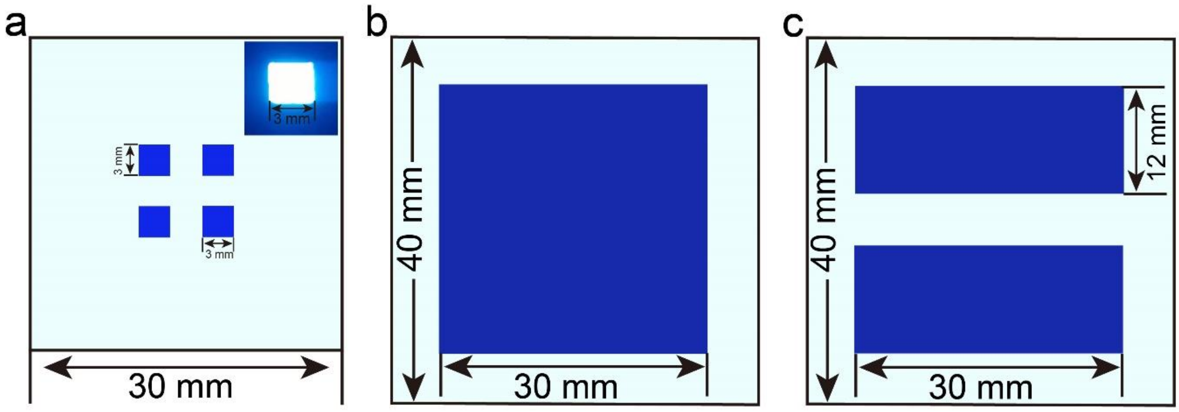

Figure S25. Schematic diagrams of the luminous areas on device. (a) The luminous areas of 3 × 3 mm^2. (b) The luminous areas of 3 × 3 cm^2. (a) The luminous areas of 1.2 × 3 cm^2.

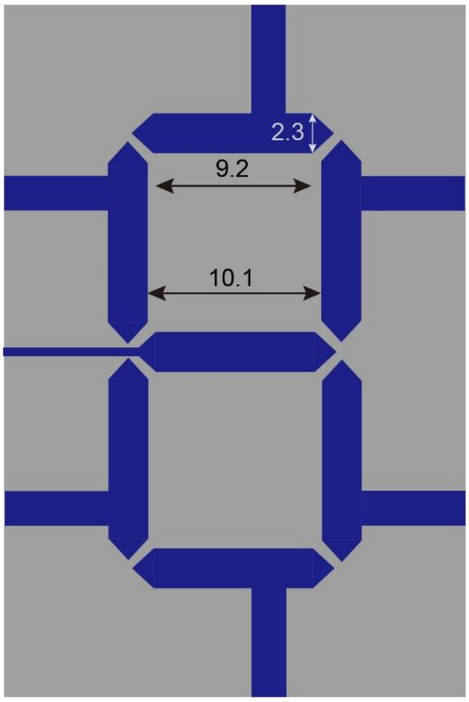

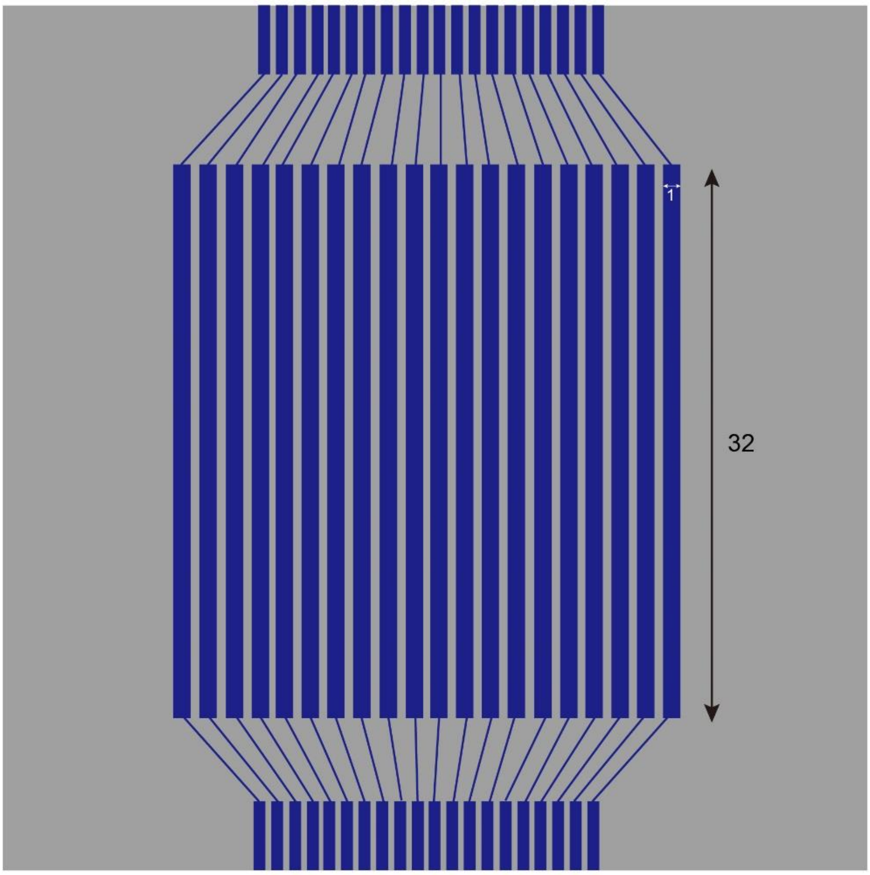

Figure S25. Schematic diagrams of template patterned array of the large-area blade-coated PLEDs.

Table S1. Summary of roughness and continuity at different temperature.

Label	Speed ·(mm/s)	Temperature ·(^{o}C)	Concentration ·(mg/mL)	Gap ·(μm)	Usage ·(μL)	Roughness	Continuity
a	15	30	10	200	80	rough	✓
b	15	50	10	200	80	smooth	✓
c	15	70	10	200	80	smooth	×

Table S2. Summary of the optimal process conditions.

Label	Speed ·(mm/s)	Temperature ·(^{o}C)	Concentration ·(mg/mL)	Gap ·(μm)	Usage ·(μL)	Thickness ·(nm)	Coating Area ·(mm^{2})	Light-emitting area ·(mm^{2})
a	15	50	10	200	60	40-45	900	9
b	15	50	10	200	80	40-45	1600	360
c	15	50	10	200	80	40-45	1600	900

Tabel S3. Summary of film thickness at different speeds.

Label	Speed ·(mm/s)	Temperature ·(^{o}C)	Concentration ·(mg/mL)	Gap ·(μm)	Usage ·(μL)	Thickness ·(nm)	Roughness	Continuity
a	5	50	10	200	80	120-130	smooth	✓
b	10	50	10	200	80	80-90	smooth	✓
c	15	50	10	200	80	40-50	smooth	✓
d	20	50	10	200	80	30-40	smooth	✓
e	30	50	10	200	80	20-30	smooth	✓

Table S4. Summary of thickness at different concentration.

Label	Speed ·(mm/s)	Temperature ·(^{o}C)	Concentration ·(mg/mL)	Gap ·(μm)	Usage ·(μL)	Thickness ·(nm)	Roughness	Continuity
a	15	50	5	200	80	20-25	smooth	✓
b	15	50	8	200	80	30-35	smooth	✓
c	15	50	10	200	80	40-45	smooth	✓
